# Revising the tectonic chronology of East–West Antarctica since the breakup of East Gondwana

**DOI:** 10.1038/s41467-026-72500-x

**Published:** 2026-04-25

**Authors:** Hakkyum Choi, Seung-Sep Kim, Sookwan Kim, Hyunggyu Choi, Yongcheol Park, Sung-Hyun Park, Fred J. Davey

**Affiliations:** 1 Division of Glacier and Earth Sciences, Korea Polar Research Institute, Incheon, Republic of Korea; 2 Department of Geological Sciences, Chungnam National University, Daejeon, Republic of Korea; 3 Ocean Climate Response & Ecosystem Research Department, Korea Institute of Ocean Science & Technology, Busan, Republic of Korea; 4 Future Technology Center, Korea Polar Research Institute, Incheon, Republic of Korea; 5 28 Cheltenham Drive, Paraparaumu, New Zealand

**Keywords:** Tectonics, Geophysics, Geodynamics

## Abstract

East–West Antarctic separation from ~43–11 Ma is well-documented through marine magnetic anomalies in the western Ross Sea, yet multiple lines of evidence suggest earlier extension, including Victoria Land uplift (~55–50 Ma) and reconstruction gaps between the Lord Howe Rise and Campbell Plateau. Here, we present marine magnetic data from the Central Basin between the Hallett Ridge and Iselin Bank revealing oceanic crust formed between Chrons 24–20 (~53–43 Ma), confirming earlier onset of East–West Antarctic motion. Forward modeling favors asymmetric extension as the preferred mechanism for forming the ~80 km wide Central Basin. This timing coincides with Transantarctic Mountains uplift and the termination of Tasman Sea spreading (~53 Ma), which redirected extensional forces southward along triple junction pathways into the Ross Sea. Our findings extend East–West Antarctic motion ~10 million years earlier than previously established, resolving the temporal discrepancy with Victoria Land uplift and reducing long-standing misfits in Southwest Pacific reconstructions.

## Abstract (web summary)

Marine magnetic data from the Ross Sea reveal that East–West Antarctic separation began ~10 million years earlier than previously recognized, reshaping tectonic models of the Southwest Pacific since Gondwana breakup.

## Introduction

The Antarctic Plate serves as a critical reference frame for global plate tectonic reconstructions, being the only major plate surrounded entirely by active spreading centers^1^. The interactions between East and West Antarctica–two distinct geological provinces that functioned as separate plates during much of the Cenozoic–directly impact our understanding of Southwest Pacific plate motions and the breakup chronology of East Gondwana. Marine magnetic anomalies in the Ross Sea preserve the record of East–West Antarctic separation, yet the timing and kinematics of early motion remain controversial. Resolving this tectonic history is essential for reducing persistent gaps in plate reconstruction models between the Lord Howe Rise and the Campbell Plateau, with implications for understanding regional tectonics and basin formation throughout the Southwest Pacific.

The breakup of Gondwana supercontinent began in the middle Jurassic (~180–160 Ma), driven by extensive plume-related magmatism that initially separated West Gondwana (South America–Africa) from East Gondwana (Antarctica–India–Australia)^2–4^. During the early stages of this fragmentation, continental rifting between East and West Gondwana produced the opening of the Weddell Sea, creating a significant marine gateway between southeastern Africa and East Antarctica^2,4^. By the late Cretaceous, deep-mantle upwelling and associated volcanism triggered the subsequent breakup of East Gondwana itself, initiating the separation of India, Australia, and Zealandia from Antarctica^5–7^. Within Antarctica, continued intra-continental extension led to the development of the West Antarctic Rift System (WARS) and the dramatic uplift of the Transantarctic Mountains, which today define the fundamental boundary between East and West Antarctica, extending from northern Victoria Land to the Weddell Sea^2–4,8^. Through these progressive tectonic episodes, Antarctica ultimately became isolated in its current polar position, followed by the later re-unification of the East and West Antarctic plates into a single Antarctic Plate^2–4^.

**Fig. 1:**
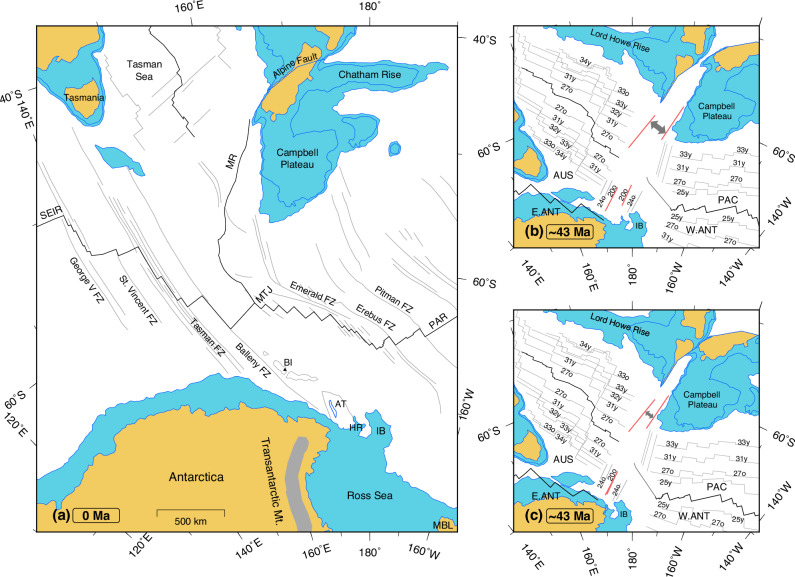
Tectonic setting of the Southwest Pacific and reconstruction models. **a** Major tectonic features of the Southwest Pacific and western Ross Sea. White areas represent oceanic crust generated by seafloor spreading. Gray lines indicate major seafloor fabric, including fracture zones^59^. Key features labeled: AT, Adare Trough; HR, Hallett Ridge; IB, Iselin Bank; BI, Balleny Islands; SEIR, Southeast Indian Ridge; PAR, Pacific–Antarctic Ridge; MTJ, Macquarie Triple Junction; MR, Macquarie Ridge Complex; MBL, Marie Byrd Land. **b, c** Comparison of two reconstruction models for the Australian–Antarctic–Pacific plate circuit at Chron 20 (~43 Ma): **b** excluding East–West Antarctic motion and **c** including East–West Antarctic motion. Red lines and gray arrows denote magnetic isochrons^60^ and the gap between the Lord Howe Rise and Campbell Plateau, respectively. Black lines show active or extinct mid-ocean ridges and tectonic offsets with transform faults^61^. Note the significant reduction in the gap between continental fragments when East–West Antarctic motion is incorporated (panel **c**). Plates labeled: AUS, Australian Plate; E.ANT, East Antarctic Plate; W.ANT, West Antarctic Plate; PAC, Pacific Plate. The reconstruction in panel **c** greatly reduces the misfit along the Australian–Pacific plate boundary that is evident in panel **b**, demonstrating the importance of East–West Antarctic motion in regional plate reconstructions.

Extensional motion between East and West Antarctica during the middle Cenozoic is well-documented through marine magnetic data from numerous studies^8–12^. Magnetic anomalies identified around the Adare Trough (AT in Fig. 1a) in the western Ross Sea confirmed that this structure represents a fossil rift valley–a key tectonic boundary resulting from extensional motion between the East and West Antarctic plates during the middle Eocene and Oligocene (approximately 43 to 26 Ma)^8^. Significantly, plate reconstruction models of the Southwest Pacific at Chron 20 (~43 Ma) that incorporate East–West Antarctic motion greatly reduce a substantial misfit along the Australian–Pacific plate boundary (i.e., the distance gap between the Lord Howe Rise and Campbell Plateau), compared to models that exclude this motion (Fig. 1b and c). More recently, the marine magnetic data collected near the northern edge of the West Antarctic Rift System (WARS) revealed continued extension during the middle Neogene, persisting until approximately 11 Ma^12^. These findings demonstrate that Antarctica functioned as two distinct tectonic plates–East and West Antarctica–from approximately 43 to 11 Ma^8,12^. Despite established evidence for East–West Antarctic separation since the middle Cenozoic, multiple lines of evidence suggest an earlier phase of motion between these plates. Previous studies^8,13^ noted this possibility because Southwest Pacific reconstruction models prior to Chron 20 (~43 Ma) consistently exhibit a significant distance gap between the Lord Howe Rise and Campbell Plateau (Fig. 1c).

**Fig. 2:**
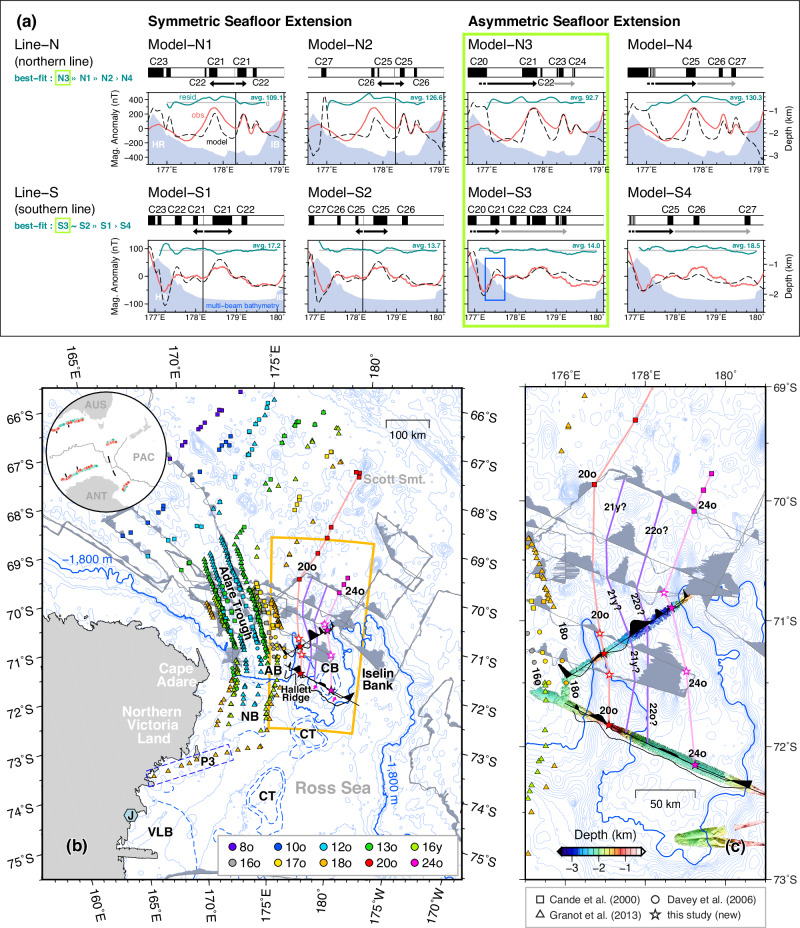
Marine magnetic evidence for Central Basin formation between Chrons 24–20. **a** Systematic comparison of symmetric and asymmetric seafloor extension models for the northern (Line-N, top) and southern (Line-S, bottom) survey lines. For each line, four models are shown: symmetric extension during Chrons 24–20 (N1/S1), symmetric extension during Chrons 27–24 (N2/S2), asymmetric extension during Chrons 24–20 (N3/S3), and asymmetric extension during Chrons 27–24 (N4/S4). Red lines show observed magnetic anomalies; black dashed lines show model predictions; pale blue regions indicate shipboard bathymetric profiles. Average residuals (avg.) are listed for each model. The best-fit ranking for each line is shown on the left. For Line-N, N3 yields the lowest residual; for Line-S, S3 and S2 produce comparable residuals, but the C27–C24 timeframe assumed by S2 is inconsistent with the observed anomaly sequence (Fig. 3). The asymmetric C24–C20 models (N3, S3; green-highlighted boxes) thus provide the most consistent fit across both lines, contradicting previous assumptions^8,13–15^. The blue box in the best model indicates the eastern flank of Hallett Ridge, where post-formation rifting or extensional event likely reduced magnetic intensity. **b** Marine magnetic picks^8,10,11^ in the Southwest Pacific Ocean and western Ross Sea, colored by age with the magnetic anomaly profiles from cruise NBP9702 (gray). Black lines (Line-N and Line-S) show survey tracks across Central Basin where Araon magnetic data were acquired. Red and pink stars indicate magnetic anomalies identified in this study (open stars from NBP9702 data; filled stars from Araon data). The yellow box area is enlarged in panel **c**. The bold blue line represents 1,800 m depth contour^62^. Dashed blue lines indicate 3–4 km sediment isopachs and Polar 3 anomaly location^11,15,23^. Key features labeled: CB, Central Basin; CT, Central Trough; AB, Adare Basin; NB, Northern Basin; VLB, Victoria Land Basin; P3, Polar 3 anomaly; J, Jang Bogo Antarctic Research Station. The inset shows magnetic picks used in rotation modeling^32^ (red and green symbols for anomalies 20 and 24; black lines for fracture zones). **c** Enlarged view showing the magnetic picks and anomaly profiles within the Central Basin. Note that NBP9702 picks (squares) can now be properly interpreted as part of the Chrons 24–20 sequence based on our complete transects.

Here, we present robust evidence for early Cenozoic motion between East and West Antarctica based on the marine magnetic data collected from the Central Basin located between the Hallett Ridge and Iselin Bank in the northwestern Ross Sea (Figs. 1 and 2). Previous magnetic surveys, including cruise NBP9702, had identified magnetic anomalies within the Central Basin but could not determine their relationship to basin formation due to incomplete coverage (Fig. 2b and c). These magnetic picks have remained in the literature for over two decades without being integrated into a coherent tectonic model. Our complete transects across the basin not only provide robust age constraints but also give these orphaned picks their proper context within the spreading history of the Ross Sea. Furthermore, our findings extend the documented history of Antarctic plate separation approximately 10 Myrs earlier than previously established, which is crucial for developing accurate Cenozoic reconstruction models for both the northern Ross Sea and the broader Southwest Pacific region. Based on these constraints, we re-examine the kinematic evolution of the Zealandia–Antarctic plate boundary since the breakup of East Gondwana, with particular focus on how the formation, migration, and evolution of triple junctions–where three tectonic plates meet–controlled the region’s complex tectonic development. This revised tectonic framework resolves long-standing inconsistencies between geological observations and plate reconstruction models, offering insights into the forces that shaped the Southern Ocean and isolated Antarctica.

## Results and discussion

### Central Basin evidencing early East–West Antarctic motion

The Central Basin was interpreted as oceanic crust based primarily on bathymetric criteria–its depth exceeding 2,000 m–though no definitive magnetic anomalies had been identified prior to our investigation^14,15^. Our marine magnetic survey across the basin reveals distinct magnetic polarity reversal patterns that confirm its oceanic crustal nature. We further integrated all available magnetic data from previous studies^8–10^, shown as different symbols in Fig. 2b, to constrain our interpretations.

**Fig. 3:**
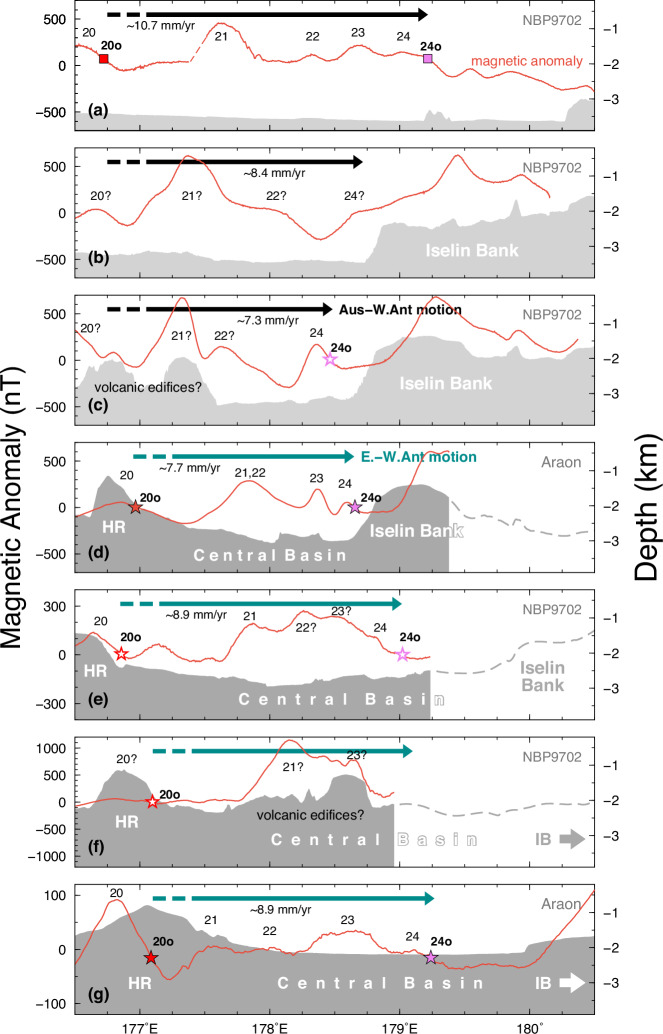
Magnetic anomaly profiles within and adjacent to the Central Basin. Seven profiles are shown, ordered from north to south. **a–c** Three NBP9702 profiles crossing Australian–West Antarctic crust north of the Central Basin (black arrows labeled “Aus–W.Ant motion” in panel **c**), providing a regional reference for the C20–C24 anomaly pattern with half-spreading rates of ~10.7, ~8.4, and ~7.3 mm/yr (north to south). In panel **a**, the dashed line represents the interpolated magnetic anomaly where the ship track was not optimal for magnetic modeling. In panel **c**, an open star marks a C24o pick from NBP9702 data near the Iselin Bank identified in this study, indicating proximity to the transition between Australian–West Antarctic and East–West Antarctic crust. **d–g** Four profiles crossing the Central Basin from the Hallett Ridge (HR) to the Iselin Bank (IB): two Araon survey lines (**d,** **g**) and two NBP9702 lines (**e,** **f**), all indicated by teal arrows labeled “E.–W.Ant motion”. Red lines show magnetic anomaly data; gray shading represents shipboard bathymetry; gray dashed lines indicate satellite-derived bathymetry where shipboard data are unavailable. Estimated half-spreading rates are labeled on each profile (~7.7 mm/yr in **d**, ~8.9 mm/yr in **e** and **g**); no rate is shown for panel **f** due to severe disruption from volcanic edifices. In panels **f** and **g**, the Iselin Bank extends beyond the plotted area (white arrows). In all Central Basin profiles (**d–g**), C20 is identified over or near the Hallett Ridge and C24 near the Iselin Bank, with a diagnostic sequence of 3–4 positive pulses (C21–C24) in between. Magnetic anomaly picks are indicated by colored symbols consistent with Fig. 2: squares for Cande et al. (2000), open and filled stars for picks identified in this study from NBP9702 and Araon data, respectively. Question marks indicate tentative identifications where amplitudes are attenuated by sediment loading or disrupted by volcanic edifices.

To evaluate the robustness of anomaly identification, we present all available profiles within and adjacent to the Central Basin, including five NBP9702 lines and our two Araon survey lines (Fig. 3). Three NBP9702 profiles (Fig. 3a–c) cross crust formed by Australian–West Antarctic motion north of the Central Basin, providing a regional reference for the expected C20–C24 magnetic anomaly pattern. The remaining four profiles — two NBP9702 (Fig. 3e and f) and two Araon (Fig. 3d and g) — traverse the Central Basin from the Hallett Ridge to the Iselin Bank and record East–West Antarctic motion. Across all profiles that cross the Central Basin, C20 is consistently identified over or near the Hallett Ridge at the western margin, and C24 near the Iselin Bank at the eastern margin. The anomaly sequence C21 through C24 comprises 3–4 diagnostic positive pulses, a pattern documented in similar-age crust of the Australian–Antarctic Basin^16^. Despite severe attenuation on Line-S due to sediment loading, this characteristic sequence is preserved and matches the patterns observed in NBP9702 data within and outside the Central Basin (Fig. 3a–c), confirming robust anomaly identification across independent datasets.

The identification of C24 as the oldest anomaly is constrained by regional context: the adjacent Hallett Ridge and Iselin Bank were positioned near Cape Adare by Chron 27^13,17–19^, and Central Basin formation postdates their emplacement, placing a lower bound on basin age. The identification of C20 as the youngest anomaly is consistent with the onset of Adare Trough spreading at Chron 20^8^, which terminated Central Basin extension, establishing an upper bound. These boundary conditions, combined with the forward modeling fit (Fig. 2a), provide robust constraints on the C20–C24 age assignment. While cruise NBP9702 partially crossed into the Central Basin and identified magnetic picks^8^, these picks were not previously linked to the basin’s tectonic history due to the lack of complete transects and definitive age constraints. Our complete transects now enable these existing NBP9702 picks to be properly interpreted within the context of the Central Basin formation.

Previously, two alternative timeframes for the formation of the Central Basin have been considered: from Chron 27 (~61 Ma) to Chron 24 (~53 Ma) or from Chron 24 to Chron 20 (~43 Ma), with extension occurring in a direction approximately parallel to seafloor formation observed in the Adare Trough^8,13–15^. Without direct magnetic evidence, the Chrons 27 to 24 (~61–53 Ma) timeframe had been favored for Central Basin formation, based primarily on plate reconstruction models that attempted to reduce the misfit between the Lord Howe Rise and Campbell Plateau along the Australian–Pacific boundary^8,13,14^. However, comparative modeling of both potential formation periods demonstrates that seafloor extension during Chrons 24 to 20 provides a significantly better fit to the observed normal and reversed-polarity bands than the previously favored hypothesis (Fig. 2a).

These findings reveal a critical revision to regional tectonic history. The adjacent Hallett Ridge and Iselin Bank, interpreted as continental fragments, were reconstructed to be positioned near Cape Adare in Victoria Land by Chron 27^13,17–19^. Chron 20 is consistently identified over or near the Hallett Ridge across multiple profiles (Figs. 2 and 3), but the ridge itself is unlikely to be entirely oceanic in origin. Its bathymetry — particularly the broader southern segment, which forms an extended shallow province at 2,000–800 m depth — suggests a composite structure comprising continental crustal fragments originally part of the East Antarctic margin, with Chron 20 oceanic crust developed at or near its eastern margin during the final stage of Central Basin extension. This interpretation is consistent with the Hallett Ridge having been positioned adjacent to the Iselin Bank at Chron 24 (~53 Ma) as a pre-existing continental feature, prior to Central Basin formation. The oceanic crust of the Central Basin then formed through extensional motion that rifted the Iselin Bank away from the Hallett Ridge and East Antarctica. In addition, the Iselin Bank occupied a critical position between major tectonic boundaries. To the north, the Nella Dan rift, and to the south, the Scott rift, bordered this continental fragment^8^. While seafloor spreading on the Southeast Indian Ridge (SEIR) east of the Balleny Fracture Zone (FZ) initiated around Chron 29 (~64 Ma)^8^, the subsequent rifting of the Iselin Bank from East Antarctica occurred significantly later, as evidenced by our identification of Chrons 24–20 spreading in the Central Basin.

This revised chronology extends the known history of Antarctic plate separation approximately 10 million years earlier than previously documented and aligns with the timing of Transantarctic Mountains uplift in Victoria Land (~55–50 Ma). Furthermore, it necessitates a reexamination of regional tectonic features that preserve evidence of early East–West Antarctic motion, particularly the Balleny FZ and Ross Sea.

### Ross Sea reconstruction since 53Ma

**Fig. 4:**
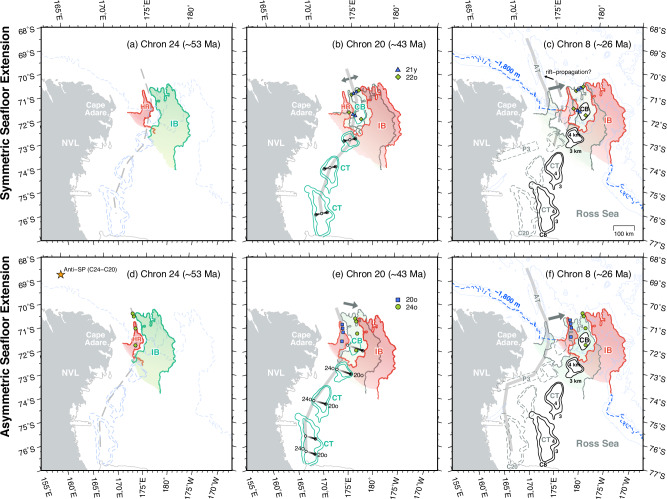
Comparison of symmetric and asymmetric seafloor extension models for the Ross Sea reconstruction. Reconstructions of the western Ross Sea at Chrons 24 (~53 Ma), 20 (~43 Ma), and 8 (~26 Ma) for symmetric (**a**–**c**) and asymmetric (**d**–**f**) extension. HR and IB are West Antarctic plate members (except HR in panels **a** and **d**). Blue dashed lines, 1,800 m depth contour^62^; black contours, present-day 3 and 4 km sediment isopachs^11,15,23^. AT, Adare Trough; CB, Central Basin; CT, Central Trough; HR, Hallett Ridge; IB, Iselin Bank; NVL, Northern Victoria Land; P3, Polar 3 anomaly. **a**–**c** Symmetric extension. **a** At Chron 24, HR and IB lie adjacent to Cape Adare prior to CB formation. The gray dashed plate boundary is reconstructed from its Chron 20 position (panel **b**) near the C21y picks, using Cande & Stock (2004)‘s C27–C24 rotation parameters. **b** By Chron 20, symmetric extension has opened CB between HR and IB, with conjugate picks (blue triangles, C21y; yellowgreen diamonds, C22o) on both flanks. CT (green outlines) develops concurrently along the strike of CB rifting; white-to-black circle pairs indicate displacement on both sides of the boundary. **c** By Chron 8, rift propagation has transferred the extensional axis from CB to AT, transferring HR to the West Antarctic plate, with subsequent ENE-WSW extension (gray arrows). **d**–**f** Asymmetric extension (preferred model). **d** As in panel **a**, but with the spreading boundary pinned against the East Antarctic lithosphere along the HR margin. Colored circles are magnetic picks reconstructed to Chron 24; the yellow star marks the antipodal stage pole (Anti-SP) for Chrons 24–20. **e** By Chron 20, ~80 km of oceanic crust has accreted on the West Antarctic side. Blue squares (C20o) and yellowgreen circles (C24o) are magnetic picks; the gray arrow shows ESE-directed IB motion. CT forms concurrently, with reconstructed 3–4 km isopachs aligning with the Victoria Land coastline at Chron 24. **f** By Chron 8, ENE-WSW extension has created AT as HR, CB, and CT move with IB as part of West Antarctica; the pinned boundary aligns with the P3 anomaly. AT boundaries in panels **c** and **f** are approximate, not derived from formal reconstruction.

Forward modeling of both symmetric and asymmetric seafloor extension scenarios (Fig. 2a) indicates that the C24–C20 age assignment and total extension of ~80 km are robust regardless of the spreading mechanism. To evaluate how different spreading geometries affect the tectonic reconstruction, we present both symmetric (Fig. 4a–c) and asymmetric (Fig. 4d–f) models below.

In order to reconstruct pre-Chron 20 seafloor spreading in the Ross Sea region using the magnetic picks identified in the Central Basin, we estimated finite rotation parameters for the East and West Antarctic plates spanning Chrons 24 to 20 (Table 1), using the global plate circuit connecting East Antarctic–Australian–West Antarctic plates (see Methods). This model forms the foundation for reconstructing the tectonic evolution of the Ross Sea during this previously unconstrained time interval.

**Table 1:** Finite rotations and covariance matrices for East–West Antarctic, East Antarctic–Australian, and Australian–West Antarctic plate motions

Magnetic Anomaly	Age^58^ (Ma)	Longitude (°E)	Latitude (°N)	Angle (degree)	Elements of Covariance Matrices								Pts.	Segs.
					$\hat{\kappa }$	a	b	c	d	e	f	g		
**E.Ant**–**W.Ant**														
20o	43.8	169.824	−56.934	8.003	0.51	8.49	−3.83	16.3	2.34	−6.72	33.4	5	N/A	N/A
24o	53.3	173.891	0.400	1.798	0.97	21.5	−1.86	60.0	3.86	−0.64	181.9	5	N/A	N/A
**E.Ant**–**Aus**														
20o	43.8	−147.620	−14.986	−24.520	0.59	1.18	−1.86	2.63	3.44	−4.87	10.8	6	85	9
24o	53.3	−145.622	−11.680	−25.297	0.77	1.60	−2.28	5.63	3.64	−8.89	89.4	6	83	7
**Aus**–**W.Ant**														
20o	43.8	−156.295	−24.303	30.034	0.43	1.76	−1.07	1.74	0.67	−1.02	1.84	4	69	10
24o	53.3	−147.995	−10.504	26.660	2.29	6.00	−3.07	8.33	1.71	−4.02	12.1	4	48	8

* The second plate (e.g., West Antarctic Plate) is fixed.

* Clockwise rotations are positive angles.

* Covariance matrix is given by this formula.

$$\frac{1}{\hat{\kappa }\,}*\left(\begin{array}{ccc}a & b & c\\ b & d & e\\ c & e & f\end{array}\right)*{10}^{-g}$$

* Pts. (Segs.) are the total number of magnetic anomaly and fracture zone points (segments) used in the calculation.

Under the symmetric model (Fig. 4a–c), the spreading axis is placed near the C21y picks within the Central Basin, with conjugate anomalies generated on both flanks. This model implies that the Hallett Ridge and Iselin Bank may have been rifted from the East Antarctic margin prior to Chron 24, with continental extension leading to oceanic crust formation in the Central Basin. During this extension, the Hallett Ridge was linked to the East Antarctic Plate. At Chron 24 (~53 Ma), both the Hallett Ridge and Iselin Bank were positioned adjacent to Cape Adare prior to Central Basin formation (Fig. 4a). By Chron 20 (~43 Ma), symmetric extension had generated the Central Basin between these features, while the Central Trough developed simultaneously to the south as an along-strike continuation of Central Basin rifting (Fig. 4b). This process parallels the mechanisms that produced the Victoria Land Basin and Northern Basin following Adare Basin formation since Chron 20 through ENE-WSW extension and subsidence in the western Ross Sea^9–11,20^. The structural continuity between the Central Basin and Central Trough — traced through gravity and seismic data with lateral offsets^15^ — is consistent with rifting that propagated southward from the Central Basin into continental crust. Subsequently, the extensional locus migrated westward into the Adare Trough, transferring the Hallett Ridge from the East Antarctic to the West Antarctic plate system (Fig. 4c). Because previous models^13^ assumed no East–West Antarctic motion during Chrons 24–20, no independent rotation parameters exist for this interval. The symmetric reconstruction shown in Fig. 4a–c therefore repurposes the Cande & Stock (2004)’s C27–C24 rotation parameters to approximate the C24–C20 extension. While internally consistent with the earlier framework, these borrowed parameters cannot be independently validated through the global plate circuit for the C24–C20 period. Furthermore, the symmetric model produces internally inconsistent spreading rates between the northern and southern profiles, and the absence of pseudo-faults argues against the intra-basin ridge jumps that would be required to reconcile these inconsistencies at the observed low spreading rates (~7–9 mm/yr).

The asymmetric model resolves these limitations. Under this model (Fig. 4d–f), the spreading boundary is interpreted as structurally pinned against the East Antarctic continental lithosphere along the Hallett Ridge margin, with oceanic crust accreting primarily toward West Antarctica. At Chron 24, both features were positioned adjacent to Cape Adare (Fig. 4d), with magnetic anomaly picks reconstructed to their Chron 24 positions, constraining the plate boundary geometry. Following Chron 24, the Iselin Bank moved in an ESE direction about an antipodal stage pole located north of Cape Adare, Victoria Land (Fig. 4d). By Chron 20 (~43 Ma), the Iselin Bank, as part of the West Antarctic Plate, had separated from the relatively stationary Hallett Ridge by approximately 80 km, with a half-spreading rate of ~8 mm/yr, generating the Central Basin between these features. As in the symmetric model, this extension simultaneously initiated the formation of the Central Trough, which connects to the southern portion of the basin (Fig. 4e). The spatial distribution of anomaly picks suggests spreading initiated in the southern Central Basin before propagating northward, consistent with rifting mechanics where extension preferentially begins near weaker continental crust before migrating oceanward^21,22^. The reconstruction is supported by seismic reflection data showing close alignment between the 3–4 km sediment isopachs^11,15,23^ of these features and the Victoria Land coastline when reconstructed to Chron 24 (Fig. 4e). The internal widths of the 4 km sediment isopachs within the Central Trough closely correspond to our calculated displacement of West Antarctica during this period. The inferred pinned boundary evolved to align with the Adare Trough and Polar 3 anomaly (Fig. 4f) as this system was abandoned at Chron 20 when the extensional axis shifted to the Adare Trough. Similar accretion asymmetry has been documented in other continent–ocean transition systems globally^24–27^, but only operated during the initial stage of continental breakup. Critically, the asymmetric model is directly constrained by C24o and C20o, the bounding isochrons identified in the Central Basin, enabling derivation of rotation parameters through the East Antarctic–Australian–West Antarctic plate circuit (Table 1). These parameters can be independently tested — a capability the symmetric model lacks. We therefore consider asymmetric extension the preferred hypothesis, while acknowledging the symmetric alternative (Fig. 2a).

Regardless of the spreading mechanism, the Hallett Ridge is best interpreted as a composite feature comprising continental crust from the East Antarctic margin with Chron 20 oceanic crust at or near its eastern margin. Under the symmetric model, the Hallett Ridge was part of the East Antarctic Plate during the Central Basin spreading phase (Chrons 24–20) and was subsequently transferred to the West Antarctic Plate when the extensional axis migrated to the Adare Trough. Under the asymmetric model, the Hallett Ridge remained relatively fixed during this interval as crust accreted primarily toward West Antarctica. Under both models, the ridge was ultimately isolated from East Antarctica at Chron 20.

Previous studies proposed a potential correlation between the Transantarctic Mountains uplift during the Cenozoic and extensional motion associated with the Adare Trough and Adare Basin formation^8,13^. However, this relationship remained uncertain due to the apparent timing mismatch between documented Cenozoic continental uplift at 55–50 Ma^3,23,28,29^ and extension episodes beginning around 43 Ma in the western Ross Sea^8^. Our model resolves this temporal discrepancy by proposing an earlier onset (~53 Ma) of East–West Antarctic motion related to Central Basin and Central Trough extension.

The extension documented in the Central Basin extends the tectonic history of East–West Antarctic motion into the early Eocene. Previous studies reported that total displacement in the Ross Sea due to East–West Antarctic separation since Chron 27 was approximately 200 km^8,13^, with well-documented components including approximately 150 km of extension in the Adare Basin (with a full-spreading rate of ~10 mm/yr)^8–11,13^, and 110 km and 95 km of extension in the Northern Basin and Victoria Land Basin (with full-spreading rates of ~7 mm/yr and ~6 mm/yr)^9–11^, respectively. These measurements left a discrepancy of at least 50 km of displacement before Chron 20 in the northern Ross Sea. Our reconstruction model addresses this gap by documenting approximately 80 km of seafloor extension in the Central Basin between Chrons 24 and 20, with a half-spreading rate of ~8 mm/yr (Fig. 4)–a rate comparable to other spreading centers in the region during this period. Based on these findings, we estimate that approximately 230 km of separation occurred in the northern Ross Sea since Chron 24 (~53 Ma), providing a more complete accounting of the displacement history between East and West Antarctica.

The Central Basin thus represents a transient tectonic system that captured the initial continental breakup between East Antarctica and the Iselin Bank during Chrons 24–20. This ~10 Myrs episode preceded the establishment of more typical seafloor spreading in the Adare Trough. The temporal correlation between Central Basin formation (~53–43 Ma) and Transantarctic Mountains uplift (~55–50 Ma) suggests a mechanical coupling between extensional tectonics in the Central Basin and vertical tectonics along the East Antarctic margin. The abandonment of this system at Chron 20 marks the transition to more conventional spreading in the Adare Trough, highlighting the Central Basin’s role as a critical but ephemeral stage in the progressive separation of East and West Antarctica. Future observations — including seismic refraction across the Hallett Ridge, dredge sampling of its basement, and higher-resolution aeromagnetic surveys — could discriminate between the asymmetric and symmetric models and further constrain the composite origin of the Hallett Ridge.

Thus, we propose that the rotation pole between East and West Antarctica gradually migrated southward into the West Antarctic Rift System (WARS) throughout the tectonic history of the Ross Sea. Our analysis places its initial position northwest of the Adare Trough (Fig. 4d) near the southern edge of the Balleny FZ, where, during Chrons 24 to 20, it facilitated the clockwise rotation of West Antarctica relative to East Antarctica. This motion created the Central Basin through primarily eastward extension while simultaneously correlating with uplift in Victoria Land. As the rotation pole subsequently shifted toward the center of the WARS following Chron 20, the extension direction changed to ENE-WSW, creating the Adare Basin, Northern Basin, and Victoria Land Basin. This progressive southward migration of the rotation pole provides a coherent framework for understanding the sequential development of different basins in the Ross Sea, explaining why each phase of extension produced structurally distinct features with different orientations throughout the region. The distinction that the asymmetric model yields rotation parameters that are independently derivable and testable, whereas the symmetric model relies on repurposed parameters from earlier intervals motivates the Balleny FZ analysis presented below.

### Balleny Fracture Zone preserving East–West Antarctic motion history

Having identified an earlier onset of East–West Antarctic motion in the Central Basin, we needed to validate these findings through independent evidence of plate motion during this period. The Balleny FZ emerges as a crucial tectonic feature that preserves the kinematic history of East–West Antarctic motion. Previous studies noted that seafloor spreading rates east of the Balleny FZ exceeded those measured between Australia and East Antarctica along the remainder of the SEIR for periods predating Chron 8 (~26 Ma)^8,13,30,31^. This spreading rate differential has been attributed to additional motion between the East and West Antarctic plates^8,13^, making the SEIR corridor east of the Balleny FZ particularly valuable for testing and quantifying our proposed early East–West Antarctic motion beginning at Chron 24.

**Fig. 5:**
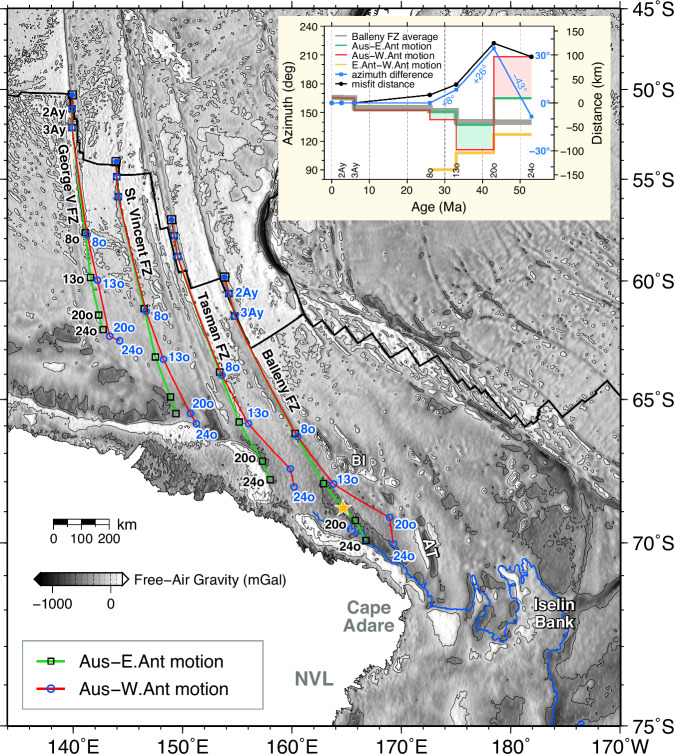
The Balleny Fracture Zone preserves East–West Antarctic motion history. Synthetic flowlines generated by backtracking arbitrary points on the eastern SEIR fracture zones. Green lines with square symbols represent flowlines produced using Australian–East Antarctic rotation parameters, while red lines with circle symbols show flowlines using Australian–West Antarctic rotation parameters. The progressive divergence between these flowlines from Chron 24 (~53 Ma) to Chron 8 (~26 Ma) demonstrates the cumulative effect of relative motion between East and West Antarctica. This divergence reaches maximum magnitude near the continental margin of Victoria Land along the Balleny Fracture Zone, providing direct evidence of sustained differential plate motion. The yellow star indicates the location of the antipodal stage pole between Chrons 24 and 20 (164.674°E, 68.880°S) derived in this study. The inset shows the azimuth difference between the East–West Antarctic motion and the Balleny FZ orientation through time. The dramatic change in azimuth difference between the Australian–East Antarctic and the Australian–West Antarctic motions during Chrons 24–20 (shaded) demonstrates the East–West Antarctic motion parallel to the FZ, explaining why mismatch does not accumulate despite ongoing extension. An azimuth difference approaching 0° indicates parallel motion (no offset accumulation), while a difference approaching 90° indicates perpendicular motion (maximum offset accumulation). Key features labeled on top of free-air gravity anomaly data^63^: AT, Adare Trough; BI, Balleny Islands; NVL, Northern Victoria Land.

To determine whether the Balleny FZ preserves evidence of early East–West Antarctic motion, we generated synthetic flowlines by backtracking arbitrary points on the eastern SEIR fracture zones for periods when relative motion between these plates existed (from anomalies 8o to 24o in Fig. 5). Using both Australian–East Antarctic and Australian–West Antarctic rotation parameters (Table 1), we observed a significant offset along the Balleny FZ, with progressive accumulation of discrepancies between respective flowlines. These discrepancies–simulated relative to the Australian Plate–reached maximum magnitude near the continental margin of Victoria Land along the Balleny FZ, providing compelling evidence of sustained differential plate motion throughout this period.

The importance of the Balleny FZ was further confirmed in our rotational modeling using the Hellinger program^32^ in conjunction with global plate circuits. All attempts to exclude the Balleny FZ or incorporate other fracture zones west of it failed to produce parameters that adequately describe East–West Antarctic motion for Chrons 24 and 20 (Fig. 5). This indicates that in the northern Ross Sea, where marine magnetic data are considerably deficient, the flowlines of the Balleny FZ represent the only preserved record that accurately captures the relative motion between East and West Antarctica during this critical time interval.

In addition, the Balleny FZ provides independent validation of East–West Antarctic motion during Chrons 24–20. Mismatch east of the Balleny FZ grows progressively until Chron 20 as previous studies documented^8^, however, it plateaus without further increase back to Chron 24 when our rotation parameters are applied. This plateau might seem inconsistent with continued plate motion, but the rotation parameters utilized in this exercise resolve this apparent paradox. The antipodal stage pole for East–West Antarctic motion during Chrons 24–20 lies along the Balleny FZ flowline itself (see yellow star in Fig. 5), causing ESE-WNW extension nearly parallel to the fracture zone flowline. As demonstrated in Fig. 5 (inset), the azimuth difference between the Australian–East Antarctic and Australian–West Antarctic motions decreased dramatically during this period, and the East–West Antarctic motion approached near-parallel alignment with the Balleny FZ orientation. This geometric configuration explains why mismatch does not accumulate from Chrons 24–20 despite ongoing extension, as the motion was oriented parallel to the fracture zone.

**Fig. 6:**
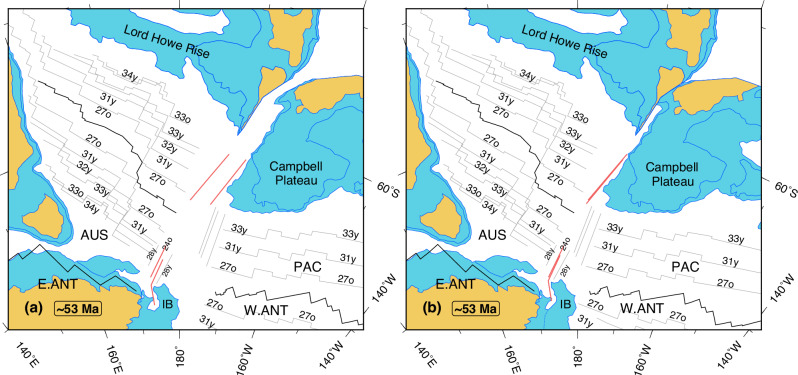
Impact of early East–West Antarctic motion on Southwest Pacific reconstruction at Chron 24. Comparison of two plate reconstructions at Chron 24 (~53 Ma): **a** Without incorporating the East–West Antarctic motion in the Central Basin, and **b** With East–West Antarctic motion included. Gray lines with labels (27o, 31y, 33y, etc.) represent magnetic isochrons^60^. Red lines highlight the misfit between continental fragments along the Australian–Pacific boundary. In panel **a**, a significant gap (~150 km) remains between the Lord Howe Rise and Campbell Plateau when using only previously documented extension. Panel **b** demonstrates that incorporating the East–West Antarctic motion with doubled rotation angle (to account for asymmetric deformation) proposed in this study greatly reduces this gap, providing a more coherent reconstruction of the Southwest Pacific region. Continental features are shown in light blue (submerged) and gold (emergent). Plates labeled: AUS, Australian Plate; E.ANT, East Antarctic Plate; W.ANT, West Antarctic Plate; PAC, Pacific Plate; IB, Iselin Bank.

Beyond validating our model locally, the asymmetric spreading mechanism proves essential for understanding the broader Southwest Pacific tectonics. Symmetric spreading in the Ross Sea would require ~80 km of additional accommodation on the East Antarctic margin, creating irreconcilable overlaps with established plate boundaries at Chron 24. The asymmetric model, however, successfully resolves long-standing reconstruction gaps between the Lord Howe Rise and Campbell Plateau (Fig. 6) while maintaining geometric consistency between the Hallett Ridge and Iselin Bank (Fig. 4).

### Revising Cenozoic reconstructions of the Southwest Pacific

Previous studies demonstrated that incorporating East–West Antarctic motion at Chron 20 (~43 Ma) substantially reduced the distance gap between the Lord Howe Rise and Campbell Plateau along the Australian–Pacific boundary^8,13^. However, a significant misfit of approximately 150 km remained in reconstructions at Chron 24 (~53 Ma) when using only previously documented extension (Fig. 6a). The East–West Antarctic separation extending back to Chron 24 identified in this study significantly reduces this remaining misfit, providing a more coherent Southwest Pacific reconstruction (Fig. 6b).

To account for the unique asymmetric deformation pattern observed in the Ross Sea, we applied a hypothesized adjustment to the rotation parameters in our Southwest Pacific reconstructions. Our data reveal that extensional forces manifested differently on either side of the tectonic boundary: producing measurable seafloor spreading toward West Antarctica while generating primarily vertical uplift rather than horizontal extension in East Antarctica’s Victoria Land. To properly account for this fundamental asymmetry, we doubled the rotation angle between East and West Antarctica when applying it to reconstructions outside the Ross Sea region. This adjustment compensates for the limitations of applying rigid plate rotation models to a region experiencing fundamentally asymmetric deformation, where one margin underwent seafloor spreading while the conjugate margin experienced presumably vertical tectonics. The effectiveness of this approach is demonstrated by our calculations: incorporating East–West Antarctic motion beginning at Chron 20 reduced the distance gap between the Lord Howe Rise and Campbell Plateau from approximately 250 km to about 120 km (Fig. 1b and c), and when the separation extending back to Chron 24 identified in this study is included with the doubled rotation angle, the remaining misfit is nearly eliminated, providing a more coherent Southwest Pacific reconstruction (Fig. 6b).

**Fig. 7:**
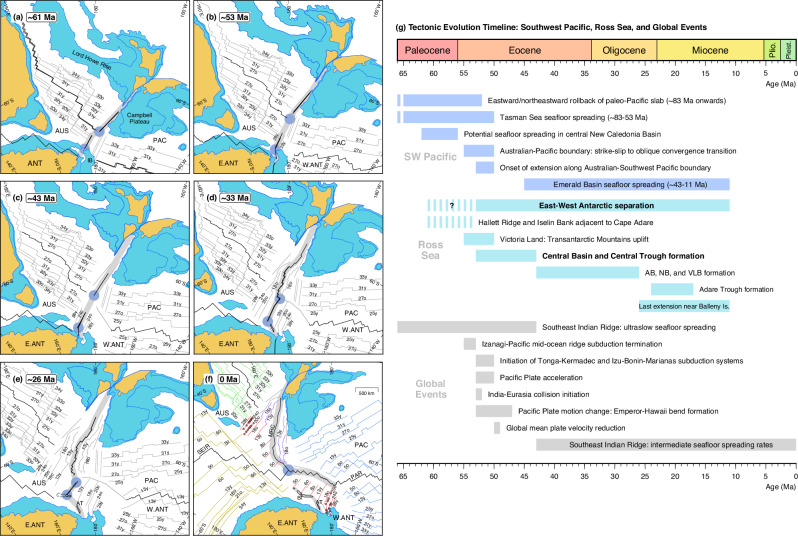
Cenozoic tectonic evolution of the Southwest Pacific region showing triple junction migration. **a–f** Time-sequential plate reconstructions from Paleocene to present: **a** At Chron 27 (~61 Ma), two critical triple junctions existed: a northern junction where the Lord Howe Rise, Australian (AUS), and Pacific (PAC) plates met, and a southern junction between the Australian, Antarctic (ANT), and Pacific plates. **b** By Chron 24 (~53 Ma), the southern triple junction evolved into a complex four-plate intersection (Australian, East Antarctic (E.ANT), West Antarctic (W.ANT), and Pacific) as Tasman Sea spreading ceased and Australian–Pacific motion changed from strike-slip to convergent. **c** At Chron 20 (~43 Ma), the Central Basin had formed between the Hallett Ridge and Iselin Bank (IB), with the tectonic configuration stabilized between an Australian–West Antarctic–Pacific triple junction in the north and an Australian–East Antarctic–West Antarctic triple junction in the south. **d** By ~33 Ma, the Adare Basin, Northern Basin, and Victoria Land Basin were developing in the Ross Sea. **e** At Chron 8 (~26 Ma), the Adare Trough (AT) had formed, with continued extension between East and West Antarctica. **f** Present-day (0 Ma) configuration showing cessation of East–West Antarctic motion and establishment of the unified Antarctic Plate. Gray lines labeled with numbers (27o, 31y, etc.) represent magnetic isochrons. Light blue areas show submerged continental fragments; gold areas show emergent land. **g** Comprehensive timeline of tectonic events in the Southwest Pacific (blue), Ross Sea (light blue), and global events (gray), highlighting temporal relationships between regional and global tectonic processes. The question mark in the East–West Antarctic separation timeline indicates the previously uncertain onset timing, which our study resolves to Chron 24 (~53 Ma).

Based on our identification of East–West Antarctic motion during Chrons 24 to 20, we have developed a revised Cenozoic reconstruction of the Southwest Pacific region that tracks the evolution of key tectonic features from Chron 27 (~61 Ma) to present (Fig. 7a). This reconstruction reveals how triple junctions–the focal points where three tectonic plates meet–controlled the sequential development of the region. At the starting point of Chron 27, two critical triple junctions existed: a northern junction where the Lord Howe Rise, Australian, and Pacific plates met (formed during Tasman Sea spreading since ~83 Ma)^33,34^, and a southern junction between the Australian, Antarctic, and Pacific plates. This southern triple junction would ultimately drive extensional forces into the Ross Sea, initiating the series of tectonic events we document in the following chronological sequence.

By Chron 24 (~53 Ma), a major global plate reorganization fundamentally altered the Southwest Pacific tectonic framework (Fig. 7b and g). This reorganization included the termination of Izanagi–Pacific ridge subduction, significant changes in absolute plate motions, decreased global plate velocities, and the initiation of the Izu–Bonin–Marianas and Tonga–Kermadec subduction systems around the Pacific Plate^35,36^. As Australian absolute motion shifted from northwest to northward, and motion along the Australian–Pacific boundary transformed from strike-slip to oblique convergence, the north-south trending Tasman Sea basin that had developed through eastward Pacific Plate rollback since ~83 Ma ceased spreading around ~53 Ma^33–35,37,38^. This pivotal change triggered the southern triple junction to evolve into a complex four-plate intersection (Australian, East Antarctic, West Antarctic, and Pacific), with extensional forces propagating southward along the line connecting the triple junctions (Fig. 7b). This extensional corridor ultimately reached the Ross Sea region, inducing the seafloor spreading we documented in the Central Basin–marking the initiation of East–West Antarctic separation.

During Chrons 24 to 20 (~53–43 Ma), the global tectonic reconfiguration stabilized as the Lord Howe Rise–previously moving as an independent plate–became incorporated into the Australian Plate by Chron 20, leaving two well-defined triple junctions in the Southwest Pacific: an Australian–West Antarctic–Pacific junction in the north and an Australian–East Antarctic–West Antarctic junction in the south (Fig. 7c). This period saw simultaneous development of the Central Basin and Central Trough in the Ross Sea along with progressive lengthening of the tectonic offset between the Tasman Sea and Southwest Pacific as the Emerald Basin began opening^39^. A critical shift occurred in the Ross Sea extensional axis from the Central Basin (between the Hallett Ridge and Iselin Bank) to the Adare Trough (between Cape Adare and the Hallett Ridge), effectively transferring the locus of extension deeper into East Antarctica while incorporating the Hallett Ridge into the West Antarctic plate system (Fig. 7c).

The Emerald Basin continued extending until approximately 11 Ma along the southern Australian–Pacific boundary^39^, eventually intersecting with the Australian–West Antarctic–Pacific triple junction (Fig. 7d and e). This extension progressively increased the gap between the Tasman Sea and the Southwest Pacific. By approximately 26 Ma, the Adare Basin, Northern Basin, Victoria Land Basin, and Adare Trough had formed within the western Ross Sea (Fig. 7d, e, and g)^8–11,19,20,40^. Concurrently, the Australian Plate–which had been slowly separating from Antarctica–accelerated northward as spreading rates increased along the SEIR after Chron 20^13^ (Fig. 7g). Although the ridge-ridge-ridge triple junction between the Australian, East Antarctic, and West Antarctic plates maintained stability throughout the 53–26 Ma period, the tectonic boundaries connecting the two triple junctions progressively lost their linear configuration as they accommodated changes in surrounding plate motions (Fig. 7e).

The East–West Antarctic separation history concluded around 11 Ma^12^, leaving its final tectonic imprints around the Balleny Islands (Fig. 7e). As these plates unified, the triple junction in the northwestern Ross Sea was incorporated into the single Antarctic Plate. The tectonic traces near the Balleny Islands represent the migration pathway of this triple junction, which followed a complex trajectory from the northern Adare Trough to the northern Balleny Islands before ceasing its function as a triple junction (Fig. 7f). Subsequently, the Macquarie Triple Junction of fault-fault-ridge configuration has maintained stability since approximately 11 Ma, coinciding with the cessation of East–West Antarctic motion.

### Triple junctions as drivers of Zealandia–Antarctic tectonic evolution

**Fig. 8:**
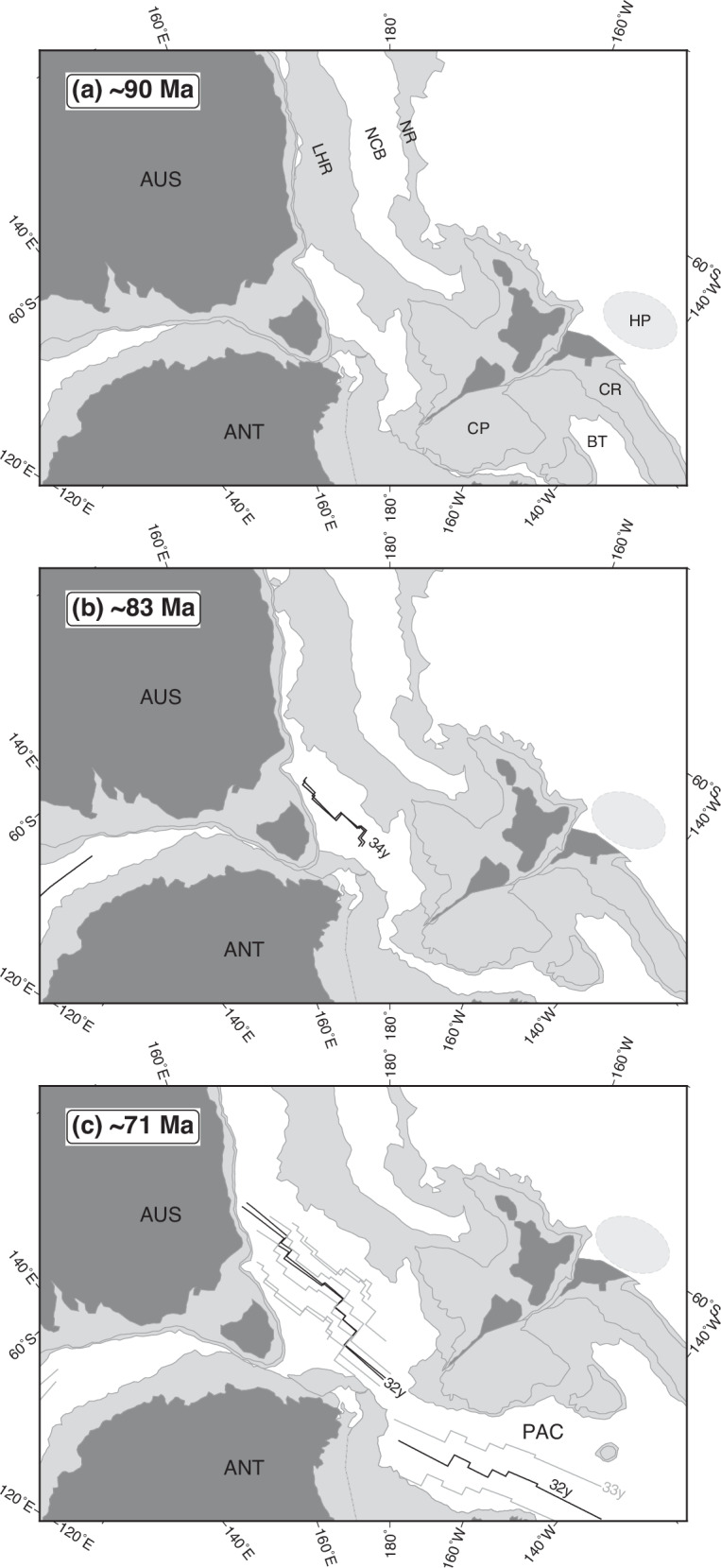
Kinematic evolution of Zealandia–Antarctic plate boundary since East Gondwana breakup. Reconstructions of the Southwest Pacific region during the late Cretaceous: **a** At ~90 Ma, East Gondwana remained largely intact, with the Lord Howe Rise (LHR) attached to Australia (AUS) and the Campbell Plateau (CP) abutting Antarctica (ANT). The Bounty Trough (BT) had already formed between the Campbell Plateau and Chatham Rise (CR) through back-arc spreading. **b** By ~83 Ma, the collision of the Hikurangi Plateau (HP) with the Chatham Rise triggered eastward/northeastward slab rollback, initiating seafloor spreading in the Tasman Sea as shown by magnetic anomaly 34. **c** At ~71 Ma, continued seafloor spreading in the Tasman Sea separated the Lord Howe Rise from Australia, while the Pacific–Antarctic Ridge began separating the Campbell Plateau from Antarctica, as evidenced by magnetic anomalies 32y and 33y. White areas represent oceanic crust; light gray areas show submerged continental fragments; dark gray areas indicate emergent land. Norfolk Ridge (NR) and New Caledonia Basin (NCB) are labeled in panel **a**. Black lines in panels **b** and **c** represent magnetic isochrons.

Building upon the East–West Antarctic motion in the Central Basin and revised Southwest Pacific reconstructions, we now present a comprehensive evolutionary model that extends from the breakup of East Gondwana in the late Cretaceous through the complete Cenozoic development of the region (Fig. 8). This synthesis integrates our findings with the oldest available rotation parameters^13,34,35^ from Chron 34 (~83 Ma) and Chron 32 (~71 Ma) to provide a cohesive tectonic framework that explains how triple junction evolution controlled regional plate dynamics.

East Gondwana–comprising Antarctica, India, Australia, and Zealandia–remained unified until the early Cretaceous. While the oldest identified marine magnetic anomaly in the region is anomaly 34 (~83 Ma)^13,34,35,41^, active mantle upwelling and extensive volcanism likely initiated East Gondwana breakup around 90 Ma^5,6,13^. During this initial phase, the Lord Howe Rise continental ribbon remained attached to Australia, with the New Caledonia Basin between the Lord Howe Rise and Norfolk Ridge forming through Cretaceous extension^37,42,43^. The Campbell Plateau in southern New Zealand abutted Antarctica’s Marie Byrd Land, while back-arc spreading had already formed the Bounty Trough between the Campbell Plateau and Chatham Rise by ~90 Ma^44^ (Fig. 8a).

A pivotal tectonic reorganization occurred around 83 Ma when the Hikurangi Plateau collided with the Chatham Rise, interrupting the southwest-directed paleo-Pacific subduction. This collision triggered eastward/northeastward slab rollback^38,45^ that initiated seafloor spreading in the Tasman Sea, separating the Lord Howe Rise from Australia^33,34,38^. Simultaneously, it terminated back-arc spreading in the Bounty Trough and initiated spreading along the Pacific–Antarctic Ridge (Fig. 8b). The alignment of anomaly 34 on the Lord Howe Rise and Australian plates confirms this continental separation was underway by ~83 Ma, while the presence of anomaly 33 in the Pacific (Fig. 8b and c) indicates that Campbell Plateau separation from Antarctica commenced contemporaneously or shortly thereafter.

**Fig. 9:**
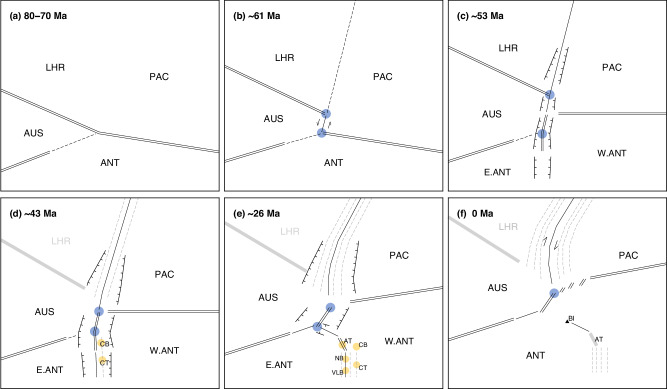
Triple junction evolution as a driving mechanism for Southwest Pacific and Ross Sea tectonics. Schematic diagram illustrating how triple junction formation, migration, and extinction controlled the sequential development of basins in the Ross Sea: **a** Late Cretaceous (80–70 Ma) configuration with initial plate boundaries established as East Gondwana began to fragment. **b** By Chron 27 (~61 Ma), a two-triple junction system had developed with the Lord Howe Rise (LHR) moving independently between the Australian (AUS), Antarctic (ANT), and Pacific (PAC) plates. **c** At Chron 24 (~53 Ma), the southern triple junction evolved into a complex four-plate intersection following Tasman Sea spreading cessation, initiating East–West Antarctic separation along an extensional corridor. **d** By Chron 20 (~43 Ma), the Central Basin (CB) and Central Trough (CT) had formed between East Antarctica (E.ANT) and West Antarctica (W.ANT), with two stable triple junctions (blue circles) controlling regional plate motion. **e** At Chron 8 (~26 Ma), ongoing triple junction migration facilitated the formation of the Adare Trough (AT), Northern Basin (NB), and Victoria Land Basin (VLB) through continued East–West Antarctic motion. **f** Present-day (0 Ma) configuration showing the unified Antarctic plate (ANT) after East–West Antarctic motion ceased (~11 Ma), with the Macquarie Triple Junction now controlling Australian–Antarctic–Pacific plate interactions. Double lines represent spreading centers; dashed lines indicate other plate boundaries; wedged lines represent extensional or rifting zones. Balleny Islands (BI) mark the final location of East–West Antarctic separation.

Critically, our reconstructions reveal that the Tasman Sea spreading ridge and Pacific–Antarctic Ridge were likely connected during this early period (Figs. 8 and 9), forming a continuous plate boundary system before the tectonic offset between them lengthened in the early Cenozoic. This interconnected boundary system explains the subsequent propagation of extensional forces into the Ross Sea region when the Australian Plate’s absolute motion changed direction during the global reorganization at ~53 Ma (Chron 24).

The Tasman Sea and Ross Sea region thus formed through related back-arc extension processes driven by paleo-Pacific slab rollback. When these extensions terminated in the early Eocene (~53 Ma) due to Australian–Pacific convergence, subsequent extensional episodes manifested through “zone jumping”–where extension migrated to structural weaknesses along pre-existing fracture zones. This mechanism explains why post-Eocene extension in the Southwest Pacific predominantly occurred along a southward-extending corridor from the Australian–Pacific plate boundary across the Zealandia continent to the Ross Sea (Figs. 7 and 9).

Our study has resolved several long-standing questions in Antarctic and Southwest Pacific tectonics. The marine magnetic evidence from the Central Basin has revealed that East–West Antarctic motion began approximately 10 million years earlier than previously recognized, at Chron 24 (~53 Ma) rather than Chron 20 (~43 Ma). This discovery resolves the temporal discrepancy between documented Transantarctic Mountains uplift (~55–50 Ma) and previously established seafloor spreading, while simultaneously eliminating reconstruction gaps between the Lord Howe Rise and Campbell Plateau in Cenozoic plate models. Our comprehensive analysis demonstrates that triple junction dynamics—their formation, migration, and extinction—served as the fundamental mechanism controlling the entire tectonic evolution of the Southwest Pacific region. The northeast-southwest trending Australian–Pacific boundary, with its migrating triple junctions, created the extensional corridor that ultimately propagated into the Ross Sea, driving the sequential formation of its basins through the Cenozoic. By integrating early Eocene motion between East and West Antarctica with this triple junction framework, we not only extend the documented history of Antarctic plate separation but also provide a complete tectonic model that coherently explains the evolution of the entire Zealandia–Antarctic plate boundary since the breakup of East Gondwana.

## Methods

### Data acquisition and processing

In 2019, we conducted marine magnetic and multi-beam bathymetric surveys across the Central Basin in the northwestern Ross Sea using the R/VIB *Araon*. Data collection focused on two survey lines crossing the basin between the Hallett Ridge and Iselin Bank (black lines, Line-N and Line-S in Fig. 2). The acquired magnetic data were processed using the International Geomagnetic Reference Field (IGRF) model 12^46^ to isolate magnetic anomalies. We then identified these anomalies through forward magnetic modeling^47^. Magnetic anomaly patterns result from the convolution of seafloor spreading rate and geomagnetic reversal intervals, producing characteristic wavelengths that can be correlated with the geomagnetic polarity timescale.

Our analysis revealed significant differences between the two survey lines. While both displayed coherent wavelength patterns indicative of seafloor spreading, Line-S showed substantially subdued intensity and predominantly negative anomaly values compared to Line-N, which exhibited both strong positive and negative peaks (Fig. 2). This difference primarily results from Line-S crossing regions with significantly thicker sediment cover (3–4 km below seafloor^11,15^) compared to Line-N. Thick sediments increase the distance between the magnetic sensor and the underlying magnetic oceanic crust, causing significant attenuation of the magnetic signal^48^.

The predominantly negative character of Line-S anomalies likely reflects a combination of factors^49–53^: the stronger attenuation of positive components from normally magnetized crustal blocks, potential thermal demagnetization from the insulating effect of thick sediments, and possible hydrothermal alteration of magnetic minerals in the underlying crust. Additionally, multi-channel seismic data reveal post-formation crustal rifting near the eastern flank of Hallett Ridge (blue box in Fig. 2a), which likely contributed to further magnetization reduction in this specific area. Despite these effects, the preservation of coherent wavelength patterns comparable to those in Line-N confirms that both survey lines crossed oceanic crust formed through the same seafloor spreading episode.

Based on these observations, our forward models employed different parameters for each survey line. For Line-N, we used a magnetized layer thickness of 0.4 km with a contamination coefficient of 0.7 (where 1 represents no contamination). For Line-S, we used a reduced magnetized layer thickness of 0.1 km and a lower contamination coefficient of 0.2 to account for the greater signal degradation. Additionally, we applied a ~135 nT positive shift to the Line-S anomalies to compensate for the systematic negative bias caused by the thick sediment cover.

To evaluate whether the observed magnetic patterns can be explained by symmetric extension, we constructed four forward models for each survey line (Fig. 2a). Models N1/S1 and N3/S3 assume the C24–C20 timeframe with symmetric and asymmetric geometries, respectively. Models N2/S2 and N4/S4 assume the previously proposed C27–C24 timeframe with symmetric and asymmetric geometries. In symmetric models, the spreading axis is located at a bathymetric high or near a magnetic high within the Central Basin, and conjugate anomalies are generated on both flanks. For the asymmetric models, crust accretes only on the eastern (West Antarctic) side. Average residuals between observed and modeled anomalies are computed for each scenario to rank the model fits (Fig. 2a). For Line-N, the asymmetric C24–C20 model (N3) yields the lowest average residual. For Line-S, the asymmetric C24–C20 model (S3) and symmetric C27–C24 model (S2) produce comparable residuals; however, the C27–C24 timeframe is inconsistent with our anomaly identification (see Results and Fig. 3). When both lines are considered jointly, the asymmetric C24–C20 models (N3, S3) provide the best overall fit.

For this final forward model, variable spreading rates were calculated for each magnetic anomaly interval. For Line-N, the estimated half-spreading rates were: 5.6 mm/yr (C24o–C23o), 7.5 mm/yr (C23o–C22o), 5.7 mm/yr (C22o–C21y), and 10.2 mm/yr (C21y–C20o), averaging 7.2 mm/yr. For Line-S: 7.9 mm/yr (C24o–C23o), 14.7 mm/yr (C23o–C22o), 5.8 mm/yr (C22o–C21y), and 6.4 mm/yr (C21y–C20o), averaging 8.2 mm/yr. The lower rates on Line-N likely reflect underestimation due to its oblique orientation relative to the spreading direction, while Line-S, oriented nearly perpendicular to anomaly strikes, provides more reliable estimates.

### Data integration and validation

All available magnetic data from the northwestern Ross Sea were incorporated and reinterpreted in our analysis (Fig. 2b). These include magnetic picks from Cande et al. (2000) (squares), Davey et al. (2006) (circles), and Granot et al. (2013) (triangles). In addition, we identified magnetic picks from NBP9702 data within the Central Basin (open stars in Fig. 2c) and from our Araon survey (filled stars). Although the NBP9702 cruise was conducted as part of the Cande et al. (2000) study, these Central Basin picks were not identified or published in that paper. The NBP9702 tracks partially entered the Central Basin but did not fully cross it, preventing definitive anomaly identification at the time. Our complete transects now provide the framework to properly interpret these previously unassigned picks as part of the Chrons 24–20 sequence (Fig. 3). The consistency between NBP9702 profiles within and outside the Central Basin and our Araon data confirms that these picks indeed record the Central Basin’s spreading history.

### Plate kinematic analysis and reconstruction

We utilized the Hellinger program^32,54^ to compute finite rotation poles and confidence ellipses describing relative plate motion between adjacent tectonic plates. Because our survey yielded magnetic data only from the West Antarctic Plate side of the boundary with no preserved conjugate anomalies on the East Antarctic side, direct calculation of rotation parameters for Chrons 24–20 was not possible. Instead, we employed a global plate circuit linking East Antarctic, Australian, and West Antarctic plates using Hellinger’s addrot program. This plate circuit calculation incorporated the magnetic data identified in this study and Balleny FZ trace with the archived magnetic anomalies and FZ traces from previous studies^8,35,55^. Following standard statistical modeling practices, we assigned uncertainties of 7 km to magnetic anomalies and 10 km to fracture zones. The resulting finite rotation poles were converted to stage poles (Figs. 4d and 5) using the rotconverter program in GMT^56^.

Because the asymmetric model assumes crust accretion primarily on one side of the boundary, the rotation parameters in Table 1 represent the total displacement observed on the West Antarctic Plate, equivalent to a half-angle rotation with respect to the full plate separation. For the Southwest Pacific reconstructions (Figs. 6 and 7), we doubled the rotation angle to account for the total displacement that would have occurred under rigid plate motion, compensating for the asymmetric deformation pattern in which one margin underwent seafloor spreading while the conjugate margin experienced presumably vertical tectonics (see Results).

Rotation parameters are expressed using the convention where positive angles represent clockwise rotation when viewed from above the rotation pole. The stage pole for Chrons 24–20 is located at 344.674°E, 68.880°N, with East Antarctica rotating 7.196° clockwise relative to fixed West Antarctica. This produces ESE-directed motion in the Central Basin region, as shown in Fig. 4d. The rotation parameters listed in Table 1 show the antipodal point of the given rotation pole to be comparable with other stage poles.

The East–West Antarctic rotation parameters derived through the plate circuit carry substantially larger uncertainties than the constituent E. Ant–Aus and Aus–W. Ant pairs (Table 1), which is an inherent consequence of resolving a small differential motion (~80 km total displacement) from the composition of two larger, independently constrained rotations^30^. For Chron 24, the near-equatorial pole location (0.4°N) and large covariance reflect the geometric reality of incipient plate motion with limited total displacement, where rotation poles are characteristically distant from the plate boundary and poorly localized^32^. Despite these formal uncertainties, the rotation parameters are independently validated by the Balleny FZ mismatch analysis (Fig. 5), which demonstrates that the derived motion direction and approximate magnitude produce predictions consistent with independently observed tectonic features. We note that the plate circuit approach ensures internal self-consistency by construction: the East–West Antarctic parameters represent the formal composition of the two well-constrained adjacent pairs, with covariance propagated through the same algebraic framework^32,54^.

For our Cenozoic reconstruction models (Figs. 6 and 7), we primarily used rotation parameters from Cande & Stock (2004), which describe Southwest Pacific evolution including the East Antarctic, West Antarctic, and Australian plates, along with the Lord Howe Rise. However, for reconstructions predating Chron 20 (~43 Ma), we substituted the parameters derived in this study to incorporate the previously unrecognized East–West Antarctic extension at Chron 24 (~53 Ma). For earlier reconstructions extending into the mid-Mesozoic (Fig. 8), we adapted rotation parameters from previous studies^13,34,35^. For the symmetric reconstruction shown in Fig. 4a–c, we repurposed Cande & Stock (2004)’s C27–C24 rotation parameters to approximate the C24–C20 extension, as their original framework assumed no East–West Antarctic motion during this interval. These parameters are not independently derived for the C24–C20 period and therefore cannot be validated through the plate circuit for that specific time interval.

## Supplementary information

Transparent Peer Review file

## Data Availability

The multibeam bathymetric and marine magnetic data generated in this study have been deposited in the Zenodo repository (10.5281/zenodo.19353249)^57^. Previously published magnetic anomaly picks are available from the cited references.
