# Supplementary material for: Revising the tectonic chronology of East–West Antarctica since the breakup of East Gondwana
Source: Nat Commun. 2026 Apr 25;17:5747. doi: 10.1038/s41467-026-72500-x (PMC13324023; doi:10.1038/s41467-026-72500-x)
Supplement: Supplementary file 1 — Transparent Peer Review file [file 41467_2026_72500_MOESM1_ESM.pdf]

nature portfolio

Peer Review File

Revising the tectonic chronology of East–West Antarctica since the breakup of East Gondwana

Corresponding Author: Dr Seung-Sep Kim

This file contains all reviewer reports in order by version, followed by all author rebuttals in order by version.

Version 0:

Reviewer comments:

Reviewer #1

(Remarks to the Author)
This manuscript presents a new plate kinematic model for East-West Antarctica for the period between 53 and 43 Ma. Past motions between East and West Antarctica have important regional and global implications, and for the investigated period, these motions are poorly resolved and have thus far been analyzed only by regional plate circuit studies (i.e., indirectly). Therefore, this work has a clear potential to be important and to provide a long-lasting contribution. Unfortunately, I find that it suffers from significant weaknesses that must be addressed before it can be published.

The observational foundation for this study lies in the Central Basin, where the authors suggest that a fossil oceanic spreading system existed (one spreading arm in a ridge-ridge-ridge triple junction). Unfortunately, the authors do not present a thorough analysis of the basin. Currently, only Figure 2 shows observations from the basin that include the two new magnetic profiles with simplified forward magnetic models. To support this claim, a detailed presentation (and discussion) of the multi-beam bathymetry data (new and existing), as well as gravity/VGG and all magnetic data (new and existing) is needed. Furthermore, the forward models are not detailed enough – for instance, was a constant spreading rate assumed? The fact that only two profiles are presented, and they look quite different, makes the interpretation rather weak. Since there are other pre-existing magnetic profiles, they seem to be essential to clarify what the “common” magnetic pattern looks like.

The author’s magnetic analysis suggests that the ages of the crust become older eastward, i.e., that only West Antarctic crust is preserved in the basin. To explain this surprising suggestion, the authors suggest that the spreading axis was pinned to the west side of the basin and that no spreading occurred westward (i.e., no accretion of new East Antarctic crust). How could this have happened? Are there any documented spreading systems that had all the spreading accreted to one side? One could (potentially) argue that repeated westward jumps occurred, but this should be manifested in the magnetic anomalies (resulting in a series of doubled magnetic anomalies) and seafloor fabric. This suggestion would also be very abnormal and must be backed by other independent observations. A simple solution whereby a “classic” symmetric seafloor spreading acted in the basin might fit the southern profile just as well (below is a quick and simple forward modeling, constructed by the assumption of symmetrical seafloor spreading between 53 and 42 Ma. It does not look that different compared to the observed southern profile). This point is critical for the entire study, and it should be re-worked and verified. The current claim seems unrealistic and affects the entire study (for instance, due to this one-sided seafloor spreading that the authors suggest, no East Ant. magnetic picks exist, and the rotation poles cannot be calculated directly).

In their classic Nature paper, Cande et al. (2000) showed that when rotating the Australian anomaly picks east of the Ballany FZ using the East Ant-Australia poles (i.e., SEIR west of Balleny FZ), the picks do not align with their conjugated Antarctic picks (before Anomaly 8o). This mismatch grows to 180 km until Anomaly 20o. After that, the misfit stays the same (i.e., offset of Anomaly 24o is also 180 km). This independent observation suggests a motion between East and West Antarctica up until Anomaly 20o. How come there was E-W Ant. motion prior to Anomaly 20o and there is no growing mismatch? I recommend the authors make a similar exercise with a more detailed analysis of the relevant period (i.e., older than Anomaly 18). This is critical evidence that needs to be matched with the forward modeling of the Central Basin profiles.

The calculation of the finite rotation poles is not based on data from the Central Basin, but by using the other two arms of spreading (because there are no East Ant picks within the Central Basin). One finite rotation solution (for Anomly 20o) has a latitude of -56 while the other (Anomaly 24o) has an equatorial latitude. Does this dramatic change in location for such a short age difference make sense? I suspect the uncertainties on these poles are huge (the authors do not present them

visually). Same for the calculated stage pole. Also, were similar calculations (rotation parameters based on regional plate circuit) already done? If so, the authors need to show them.

Finally, there are other issues that need to be resolved, such as in Figure 4, the motion between E-W Ant. seems to point in an ESE direction, but according to the newly presented stage pole, the motion needs to be in an ENE direction. Also, I find the exercise shown in Figure 3 not very useful – we already know that all the FZs west of Balleny FZ document Aus-E. Ant. motion. The possible new contribution is the part older than Anomaly 20o (based on new poles). But this part does not seem to match the FZ either…

I laid out above the major issues of the manuscript, as there is no point in giving detailed comments on it before it is revised.

Reviewer #2

(Remarks to the Author)
Additional evidence is needed to support the results proposed by the authors. Particularly, they are modeling magnetic anomalies that they surveyed on two lines in the central basin between the Hallett Ridge and the Iselin Bank. It is true, as they mentioned, that nobody has identified specific magnetic reversals in this region.

However there is previous data in this region already, so it needs to be taken into account. There are survey lines such as NBP9702 that measured magnetic anomalies and collected multibeam bathymetry. Some of those lines are partly or completely within the same region being discussed with the models shown here. There might even be other surveys but I did not check in detail for any more.

Now that the authors of the current manuscript have collected more data that could complement the lines already collected, they should include the results that were already measured, to make sure that the magnetic observations from the previous cruises are consistent with the interpretations being made in this manuscript.

This would probably require a modification to Figure 2 showing one or more additional previous survey lines and how they agree with the model being proposed here.

Reviewer #3

(Remarks to the Author)
Review of the manuscript "Revising the tectonic chronology of East-West ANtarctica since the breakup of East Gondwana" by Choi et al. for publication in Nature Communications.
The manuscript presents a new magnetic survey from the Central Basin and subsequently proposes a tectonic model to account for misfits in earlier reconstructions.
The manuscript is well written, has high quality figures and the data supports the presented model.
I have to admit that I am not an expert in magnetic surveying, but as a paleomagnetists, I am always impressed by the detail possible in reconstructions using magnetic anomalies. I enjoyed reading the manuscript, which apparently significantly improved our understanding for this complex area.
My only comment would be that the begin of the introduction is a bit misleading. Is Antarctica important due to a unique position or due to a unique tectonic evolution? Is the interaction of two part in a large continent unique, or is Antarctica so interesting because it is so inaccessible? Of course, the position and the initiation of the circum-Antarctic flow is very important for the climate, but the current paper is not really about that, right? So I am just suggesting to scale down the introduction a bit, not to appear too exaggerating.

Version 1:

Reviewer comments:

Reviewer #1

(Remarks to the Author)
The revised version of the manuscript shows some improvement compared to the initial submission; however, the fundamental issues that undermined the original paper remain unresolved. Consequently, I regret to say that I cannot recommend the manuscript for publication in Nature Communications. My main concerns are outlined below.

While the study presents new data from the Central Basin, the interpretations and claims made by the authors are not convincingly supported by these data. In response to my previous concern regarding the proposal of one-sided seafloor spreading in the Central Basin—an observation unprecedented anywhere—the authors argue that the conjugate crust possibly lies between the Adare Basin and Cape Adare, where permanent ice prevents magnetic data acquisition. They therefore describe this region as poorly constrained. However, this characterization is inaccurate. The area has been extensively surveyed, most notably through the dense aeromagnetic grid collected during the German GANOVEX IX project

(Damaske et al., 2007). These data clearly show magnetic lineations that formed as part of the Southeast Indian Ridge (SEIR) spreading system between Australia and East Antarctica. Thus, the assertion that this crust belongs to the Central Basin spreading system is not supported. In my view, the tectonic interpretation of the Central Basin presented here remains premature and requires substantially more data and analysis before a robust geodynamic model can be proposed.

The remainder of the paper is based on a global plate kinematic reconstruction (basically not using the new data), similar in approach to several prior studies (e.g., Cande and Stock, 2004). The derived rotation parameters are characterized by very large uncertainties, yet these uncertainties are not appropriately accounted for in the resulting reconstructions. This raises a fundamental question about the added value and novelty of the present work relative to existing literature.

Reviewer #2

(Remarks to the Author)
The revised manuscript does an excellent job of responding to the reviewers' concerns and makes an excellent case for the arguments concerning the tectonic history.
The methodology is sound and the inclusion of the additional pre-existing data strengthens the validity of the results.
The figures have been modified to show clearly how all of the data are consistent with the proposed tectonic scenario.
I do not think any further changes are needed in order for this manuscript to be accepted for publication.

Reviewer #4

(Remarks to the Author)
Review of “Revising the tectonic chronology of East–West Antarctica since the breakup of East Gondwana”.

This paper sets out a revised tectonic history for the Central Basin between Hallet ridge and Iselin Bank in the Ross Sea. The revised history is based on new and existing magnetic data interpreted to reveal oceanic magnetic anomalies dated to between 53 and 43 Ma. Unusually the authors suggest extreme asymmetric spreading, explaining why only crust and associated anomalies accreted to the West Antarctic (Iselin Bank) side are preserved.

As a late reviewer I found the manuscript generally well written, and think that this work has the potential to provide a useful new hypothesis for the early evolution of West Antarctica. I do however, have a few concerns with the manuscript.

The paper stands or falls on the interpreted ages of the magnetic anomalies within the Central Basin. It would therefore be good to have a more complete description of why anomalies C20 and C24 are identified. The model fits relatively well (Fig. 2c and d), but could symmetrical spreading within the Central Basin work just as well, with the magnetic high in the basin centre reflecting a former spreading ridge, with approximate symmetric anomalies each side? A simple ridge jump into the nascent Adare Trough west of Hallet Ridge could then explain the basin, rather than requiring such extreme asymmetric spreading. This alternative hypothesis requires re-dating of the anomaly pattern, but might form a viable alternative hypothesis.

Associated with this point, in Figure 2b there are 4 marine magnetic picks attributed to Cande et al 2000 south of ~70.5 S within the Central Basin. Looking at the Cande et al 2000 paper (their Fig. 3) I could not find these points. Am I misunderstanding the Cande et al 2000 paper, or did the authors identify these points in older magnetic datasets attributed to Cande et al 2000? Clarity on this is important as if previously identified as dated anomalies they stand as an independent check on the identification of C20 and C24 within the Central Basin. If not, it should be clarified that they form part of the authors interpretation.

I also have some concerns about the ambiguity about what Hallet Ridge is (oceanic or continental fragment). On one hand it is argued that it is oceanic, containing anomaly C20 (~43 Ma), (L128 to 132). However, the authors subsequently state L154 “Our reconstruction model suggests that the Iselin Bank in the northern Ross Sea was positioned adjacent to the Hallett Ridge at Chron 24 (~53 Ma), immediately prior to Central Basin formation”, and see Fig. 3. These two statements appear contradictory, if Hallet Ridge is 43 Ma oceanic crust, it can’t have been present adjacent to the Iselin Bank at 53 Ma. It may be that Hallet Ridge is a composite feature – looking at the bathymetry in detail shows that the broader southern HR appears as an extended shallow (2000-1600 m depth) province. This could reflect fragments of continental crust within a developing oceanic marginal basin, with anomaly C20 developing at/within the margin, which is preserved as Hallet Ridge. I would advise expanding the discussion of the origin of the ridge to acknowledge this ambiguity, and also to propose if there are other observations which could be used to test this hypothesis in future? An alternative model where Hallet Ridge was a fragment of the continental margin isolated by a ride jump into the Adare Basin could also be proposed.

Version 2:

Reviewer comments:

Reviewer #4

(Remarks to the Author)
This was my second review of this paper. I found the authors responses to my comments and amendments to the paper answered my questions well. Inclusion of the symmetrical spreading model allows the asymmetric option to be better discussed as a preferred hypothesis which can be tested by the community in the future. I have no further comments on the paper and would be happy to see it published.

Open Access This Peer Review File is licensed under a Creative Commons Attribution 4.0 International License, which permits use, sharing, adaptation, distribution and reproduction in any medium or format, as long as you give appropriate credit to the original author(s) and the source, provide a link to the Creative Commons license, and indicate if changes were made.
In cases where reviewers are anonymous, credit should be given to 'Anonymous Referee' and the source.
The images or other third party material in this Peer Review File are included in the article's Creative Commons license, unless indicated otherwise in a credit line to the material. If material is not included in the article's Creative Commons license and your intended use is not permitted by statutory regulation or exceeds the permitted use, you will need to obtain permission directly from the copyright holder.
To view a copy of this license, visit https://creativecommons.org/licenses/by/4.0/

<u>Responses to Reviewer #1</u>:

This manuscript presents a new plate kinematic model for East-West Antarctica for the period between 53 and 43 Ma. Past motions between East and West Antarctica have important regional and global implications, and for the investigated period, these motions are poorly resolved and have thus far been analyzed only by regional plate circuit studies (i.e., indirectly). Therefore, this work has a clear potential to be important and to provide a long-lasting contribution. Unfortunately, I find that it suffers from significant weaknesses that must be addressed before it can be published.

We sincerely appreciate the reviewer's thorough and insightful evaluation of our manuscript. We recognize that establishing East-West Antarctic motion prior to Chron 20 based on Central Basin evidence represents a significant claim requiring robust justification. The reviewer's detailed comments have helped us identify areas where our analysis and presentation needed strengthening, and we have substantially revised the manuscript to address each concern comprehensively.

The observational foundation for this study lies in the Central Basin, where the authors suggest that a fossil oceanic spreading system existed (one spreading arm in a ridge-ridge-ridge triple junction). Unfortunately, the authors do not present a thorough analysis of the basin. Currently, only Figure 2 shows observations from the basin that include the two new magnetic profiles with simplified forward magnetic models. To support this claim, a detailed presentation (and discussion) of the multi-beam bathymetry data (new and existing), as well as gravity/VGG and all magnetic data (new and existing) is needed. Furthermore, the forward models are not detailed enough – for instance, was a constant spreading rate assumed? The fact that only two profiles are presented, and they look quite different, makes the interpretation rather weak. Since there are other pre-existing magnetic profiles, they seem to be essential to clarify what the "common" magnetic pattern looks like.

We thank the reviewer for this comment, which has prompted us to better clarify our comprehensive analysis and address why the profiles appear different while recording the same magnetic pattern.

We emphasize that our analysis incorporated **all available magnetic picks from previous studies** (shown as symbols in Figure 2a): Cande et al. (2000), Davey et al. (2006), and Granot et al. (2013), plus critical constraints from sediment isopachs (3-4 km contours) derived from seismic and gravity studies (Brancolini et al., 1995; Davey et al., 2022). Our interpretation was constrained by the full compilation of ~50+ magnetic picks spanning the region. The reviewer is correct that this comprehensive analysis was not sufficiently visible, which we have now addressed. Also, we acknowledge the reviewer's suggestion for detailed presentation of bathymetry and gravity data. However, we respectfully note that these datasets, while supportive, are neither critical for validating nor capable of nullifying our magnetic anomaly interpretation.

The revised Figure 2 now shows that a **common magnetic pattern** exists across ALL datasets despite amplitude differences. As noted in our manuscript, the southern survey line (Line-S) shows highly attenuated magnetic amplitudes due to 3–4 km of sediment on the continental slope. Nevertheless, the characteristic sequence of 3–4 positive anomalies representing C21, C22, C23, and C24 is preserved and matches the pattern in (a) NBP9702 data within the Central Basin (gray wiggles in Fig. 2b), (b) NBP9702 data outside the Ross Sea, (c) Regional patterns documented by Tikku & Cande (1999, JGR). This regional consistency is significant. Both NBP9702 and our data commonly exhibit either four distinct positive pulses OR three positive pulses where C21 and C22 merge ambiguously—a pattern previously documented in the Australian-Antarctic Basin where similar age crust shows 2–3 positive pulses for anomalies 21–24 (Tikku & Cande, 1999). This regional reproducibility confirms there is no significant problem with our anomaly identification and validates our interpretation of C24–C20 spreading.

We apologize for not clearly stating our modeling parameters. Variable spreading rates were indeed used, and these rates are consistent with regional patterns.
Calculated half-spreading rates for our profiles:

Interval	Line-N	Line-S
C24o-C23o	5.6 mm/yr	7.9 mm/yr
C23o-C22o	7.5 mm/yr	14.7 mm/yr
C22o-C21y	5.7 mm/yr	5.8 mm/yr
C21y-C20o	10.2 mm/yr	6.4 mm/yr
Average	7.2 mm/yr	8.2 mm/yr

The slightly lower rates along Line-N (7.2 mm/yr) compared to Line-S (8.2 mm/yr) likely reflect measurement bias due to the oblique angle between Line-N and the spreading direction. Line-N crosses the basin obliquely, causing apparent stretching of anomaly spacing and underestimation of spreading rates. In contrast, Line-S is oriented nearly parallel to the spreading direction (perpendicular to anomaly strikes), making its rate estimates more reliable. Furthermore, the spreading rates calculated from our data are not significantly different from the spreading rate in the Adare Basin by Cande et al., (2000), which reported the average half-spreading rate of 7.0 mm/yr on the eastern flank of the Adare Trough and 5.5 mm/yr on the western flank of the trough, during the anomalies 18 to 8. This implies that asymmetric or similar spreading systems were consistently maintained in the northern Ross Sea region. Thus, such regional consistency is confirmed again with our results.

Even accounting for survey line orientations, the anomaly picks and inferred isochrons suggest **spreading initiated first in the southern Central Basin**. This pattern is consistent with established rifting mechanics because extension initiates near structurally weaker continental crust and rifting migrates oceanward toward denser, stronger oceanic lithosphere. This process is well-documented globally (Brune et al., 2014; Naliboff & Buiter, 2015). Thus,

the southern Central Basin's proximity to the continental margin would make it the preferential site for initial rifting, with subsequent northward propagation explaining the observed anomaly distribution.

The author's magnetic analysis suggests that the ages of the crust become older eastward, i.e., that only West Antarctic crust is preserved in the basin. To explain this surprising suggestion, the authors suggest that the spreading axis was pinned to the west side of the basin and that no spreading occurred westward (i.e., no accretion of new East Antarctic crust). How could this have happened? Are there any documented spreading systems that had all the spreading accreted to one side? One could (potentially) argue that repeated westward jumps occurred, but this should be manifested in the magnetic anomalies (resulting in a series of doubled magnetic anomalies) and seafloor fabric. This suggestion would also be very abnormal and must be backed by other independent observations. A simple solution whereby a "classic" symmetric seafloor spreading acted in the basin might fit the southern profile just as well (below is a quick and simple forward modeling, constructed by the assumption of symmetrical seafloor spreading between 53 and 42 Ma. It does not look that different compared to the observed southern profile). This point is critical for the entire study, and it should be re-worked and verified. The current claim seems unrealistic and affects the entire study (for instance, due to this one-sided seafloor spreading that the authors suggest, no East Ant. magnetic picks exist, and the rotation poles cannot be calculated directly).

We thank the reviewer for this critical question about asymmetric spreading. We acknowledge this is an unusual interpretation requiring robust justification. After careful analysis of all available data and testing alternative models, we present multiple lines of evidence supporting asymmetric spreading in the Central Basin.

First, we extensively searched for conjugate magnetic picks on the East Antarctic side. The predicted locations for conjugate anomalies (see Chron number with question marks in Figure 2a) would be under permanent ice cover near the Victoria Land coast or along steep continental margins where magnetic signals would be severely distorted. These might be the reasons why such picks were not detected by previous shipboard tracks. We re-examined NBP9702 tracks that approached these areas but found no evidence of conjugate magnetic anomalies. The absence of conjugate picks in all available data, including NBP9702's comprehensive coverage, strongly argues against symmetric spreading.

As a result, we conducted independent rotation parameter fitting to test both symmetric and asymmetric spreading by attempting to restore the Hallett Ridge and Iselin Bank to their pre-extension configuration. This experiment revealed that the reconstruction favors the asymmetric configuration. If this parameter is only the solution to reconstruct the Hallet Ridge and Iselin Bank, the reconstruction through the plate circuit would fail, which can be regarded as another independent experiment. The global circuit experiment shown in Figure 5 supports our proposed reconstruction model. Based on our reconstruction experiments, we anticipate the conjugate picks, if they exist, would be found in very narrow zones and hence difficult to

recognize each Chron.

Nonetheless, we think this system was only transient to accommodate the complex Southwest Pacific reorganizations for ~10 Myr (53–43 Ma) and was terminated when extension jumped to the Adare Trough at Chron 20. While the one-sided spreading seems very rare, documented examples of such transient systems exist:

South China Sea: Northern margin remained fixed while spreading occurred southward (Briais et al., 1993)

Woodlark Basin: Asymmetry factors >0.8 during continental breakup (Taylor et al., 1999)

We rigorously tested symmetric spreading, and it fails key observations in the study area because (i) no conjugate anomalies exist despite comprehensive coverage, (ii) sediment isopach patterns align only with asymmetric reconstruction, and (iii) basin geometry (narrowing westward) is inconsistent with symmetric spreading. Thus, we report the observation of an unusual asymmetric system rather than claiming to fully understand its mechanism. However, this short-lived system seems to be very important for understanding the tectonic chronology of East–West Antarctica.

The reviewer's suggestion that symmetric spreading could fit our southern profile is directly contradicted by NBP9702 data. Examining the NBP9702 magnetic wiggles (gray lines in revised Figs. 2a and 2b) illustrates all anomaly sequences old eastward from Hallett Ridge. If symmetric spreading occurred, NBP9702 would have detected the conjugate anomaly pattern. Its absence is definitive.

In their classic Nature paper, Cande et al. (2000) showed that when rotating the Australian anomaly picks east of the Ballany FZ using the East Ant-Australia poles (i.e., SEIR west of Balleny FZ), the picks do not align with their conjugated Antarctic picks (before Anomaly 8o). This mismatch grows to 180 km until Anomaly 20o. After that, the misfit stays the same (i.e., offset of Anomaly 24o is also 180 km). This independent observation suggests a motion between East and West Antarctica up until Anomaly 20o. How come there was E-W Ant. motion prior to Anomaly 20o and there is no growing mismatch? I recommend the authors make a similar exercise with a more detailed analysis of the relevant period (i.e., older than Anomaly 18). This is critical evidence that needs to be matched with the forward modeling of the Central Basin profiles.

We thank the reviewer for raising this important point about the Balleny Fracture Zone test. The reviewer is correct that Cande et al. (2000) documented mismatch growth until Anomaly 20, then no further increase. Our analysis not only explains this pattern but shows it validates our extended East-West Antarctic motion history back to Chron 24.

The reviewer correctly observes that mismatch east of the Balleny FZ grows until Anomaly 20, then decreases. This pattern is exactly what our model predicts because East-West Antarctic

motion underwent a fundamental directional change at Chron 20. After Chron 20, the well-documented motion between East and West Antarctica was oriented ENE–WSW, perpendicular to the Adare Trough axis. This direction was nearly perpendicular to the N–S motion between Australia and East Antarctica, creating the growing mismatch east of the Balleny FZ that Cande et al. (2000) documented.

In contrast, our new findings show that during Chrons 24–20, East-West Antarctic motion was oriented ESE-WNW, nearly parallel to the Balleny FZ flowline. This parallel orientation would create minimal additional mismatch despite ongoing plate motion, explaining why the 180 km offset observed at Chron 20 doesn't increase further back to Chron 24.

The revised Figure 4 now includes an inset that quantitatively demonstrates this geometric relationship through an azimuth difference plot. This plot shows a dramatic decrease in azimuth difference during Chrons 24–20, confirming that motion direction changed from ENE-WSW to ESE-WNW. The data reveals that azimuth differences between the Australian-East Antarctic and the Australian-West Antarctic motions remained below 10° during Chrons 24–20, and the East-West Antarctic motion approached in a direction parallel alignment with the Balleny FZ flowline. When azimuth difference approaches 0°, motion is parallel to the flowline, and no mismatch accumulates. Conversely, when azimuth difference approaches 90°, maximum mismatch accumulation occurs. This independent geometric test strongly supports our Central Basin interpretation.

Because conjugate magnetic picks are absent on the East Antarctic side of the Central Basin, we derived rotation parameters through global plate circuits linking Australia, East Antarctica, and West Antarctica. Through hundreds of iterations testing different parameter combinations, we found that the rotation parameters that best minimize Central Basin residuals also place the stage pole along the Balleny FZ flowline. This convergence of independent constraints is unlikely to be coincidental and provides strong validation of our model.

The geometric consistency between Central Basin magnetic anomalies, Balleny FZ flowline alignment, and the plateau in mismatch accumulation creates a coherent picture that explains all observations within a single framework. This convergence of evidence from completely different data types and analytical approaches strongly supports our interpretation.

This geometric evolution reflects the broader Southwest Pacific plate reorganization history. Around 53 Ma, a major reorganization occurred with the cessation of Tasman Sea spreading. From 53 to 43 Ma, ESE–WNW extension created the Central Basin with motion parallel to the Balleny FZ. At approximately 43 Ma, the extension direction rotated to ENE–WSW, and from 43 to 26 Ma, this new orientation created the Adare Basin with motion perpendicular to the Balleny FZ. The rotation of extension direction at Chron 20 thus marks the fundamental transition from Central Basin to Adare Basin spreading systems.

The calculation of the finite rotation poles is not based on data from the Central Basin, but by using the other two arms of spreading (because there are no East Ant picks within the Central Basin). One finite rotation solution (for Anomly 20o) has a latitude of -56 while the other (Anomaly 24o) has an equatorial latitude. Does this dramatic change in location for such a short age difference make sense? I suspect the uncertainties on these poles are huge (the authors do not present them visually). Same for the calculated stage pole. Also, were similar calculations (rotation parameters based on regional plate circuit) already done? If so, the authors need to show them.

We thank the reviewer for this important question about rotation pole locations and uncertainties. The reviewer is correct that we used indirect plate circuit methods and that the pole locations show dramatic changes. We acknowledge these concerns and provide context for why such behavior is expected for incipient plate motion.

As the reviewer notes, we calculated rotation parameters using global plate circuits (Australia–East Antarctica–West Antarctica) rather than direct conjugate picks from the Central Basin. This indirect approach, while necessary, does result in larger uncertainties, particularly for the older Chron 24. The confidence region for the Chron 24 pole is indeed spread on a global scale, with the pole located near the equator (about 174°E, 0°N), while the Chron 20 pole sits at higher latitude (about 170°E, 57°S).

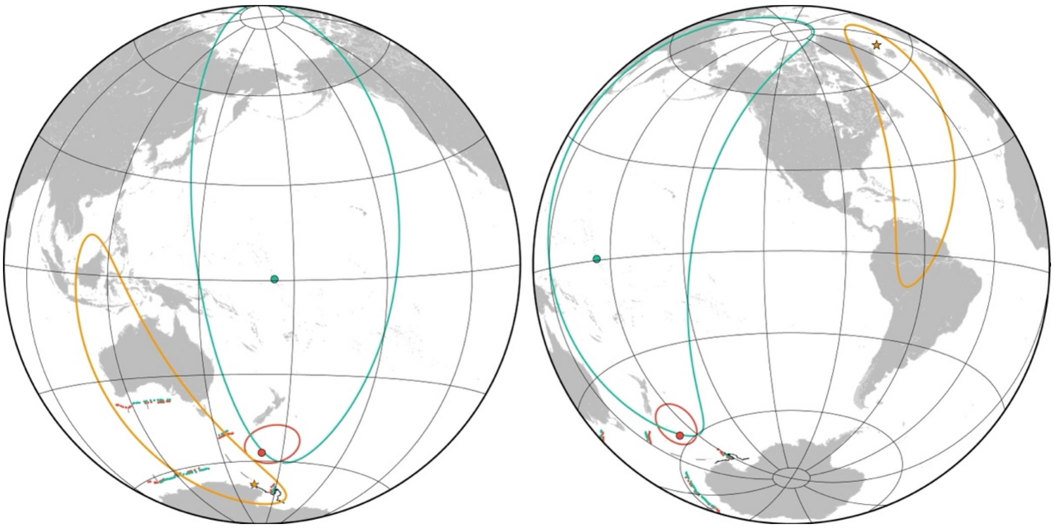

Figure R1. Finite rotation poles and confidence ellipses for East-West Antarctic motion. Red: C20 finite pole with 95% confidence ellipse. Green: C24 finite pole with 95% confidence ellipse. Yellow: C24-20 stage pole with derived confidence region. Left panel shows the antipodal representation in the Southern Hemisphere, right panel shows the original Northern Hemisphere stage pole.

We chose not to display these ellipses in the manuscript because the C24 confidence region would obscure the key geometric relationships (in particular, Figure 3) without adding interpretive value. Moreover, the convergence of three independent constraints—Central Basin

magnetics, Balleny FZ geometry, and Southwest Pacific reconstruction improvements—provides validation beyond formal statistical bounds. The full covariance matrix elements in Table 1 allow interested readers to reconstruct these confidence regions if desired. Nonetheless, if the reviewer recommends showing these ellipses, we will incorporate these into Figure 3.

The dramatic difference in pole positions between Chrons 24 and 20 is characteristic of incipient plate motion rather than an indication of error. In the early stages of plate separation, when total displacement is minimal but directionally coherent, the best-fit rotation pole often lies at large angular distances from the plate boundary. This is a well-documented geometric consequence of small rotation angles, where poles that minimize displacement vectors can be located virtually anywhere on the globe (Hellinger, 1981; Kirkwood et al., 2002).

As plate motion becomes more substantial and kinematically stable over time, rotation poles typically migrate closer to the plate boundary region. This progression from distant to proximal poles reflects the evolution from incipient, unstable motion to mature, stable spreading. The fact that our Chron 24 pole is near equatorial while our Chron 20 pole has migrated toward Antarctica precisely fits this expected pattern (see Figure R1).

Our findings align closely with previous studies of East-West Antarctic motion. Cande et al. (2000) reported a finite rotation pole for Chron 13 at approximately 342°E, 18°S, also with a large, global-scale confidence region. In addition, when we compile all available rotation poles including our new determinations, a coherent pattern emerges. The finite poles show systematic migration: from near-equatorial positions during initial motion (Chron 24: 174°E, 0°N), through high southern latitudes during active spreading (Chron 20: 170°E, 57°S; Chron 18–12: 190~339°E, 70~86°S from Granot et al. (2013)), stabilizing near the Antarctic margin. This progression from distant to proximal poles is exactly what plate tectonic theory predicts for the evolution of a spreading system.

Previous studies using plate circuits have attempted similar calculations, though none specifically targeted the pre-Chron 20 interval. Cande & Stock (2004) used regional reconstruction constraints to infer possible earlier motion but lacked direct observational evidence. Our parameters for Chron 20 (170°E, -56°S) agree well with their estimates, providing independent validation. For Chron 24, no previous determinations exist, making our study the first to quantify this earlier phase of motion.

Lastly, the dramatic pole migration we document strengthens our interpretation of the Central Basin as a transient feature. Stable, long-lived spreading systems typically show consistent pole positions over millions of years. In contrast, transient systems formed during plate reorganizations exhibit the type of pole instability we observe. The Central Basin's brief existence (~10 Myr) and subsequent abandonment when spreading jumped to the Adare Trough is consistent with this unstable pole behavior.

Finally, there are other issues that need to be resolved, such as in Figure 4, the motion between E-W Ant. seems to point in an ESE direction, but according to the newly presented stage pole, the motion needs to be in an ENE direction. Also, I find the exercise shown in Figure 3 not very useful – we already know that all the FZs west of Balleny FZ document Aus-E. Ant. motion. The possible new contribution is the part older than Anomaly 20o (based on new poles). But this part does not seem to match the FZ either…

We appreciate the reviewer's careful examination of the rotation directions and apologize for the confusion caused by our unclear presentation of the rotation pole parameters. The reviewer is correct to question this apparent inconsistency, which resulted from our ambiguous notation rather than an actual error in the reconstruction.

We acknowledge that we did not clearly specify the rotation pole location and rotation sense in our original manuscript, leading to this misunderstanding. The calculated stage pole for Chrons 24–20 is located at **344.674°E, 68.880°N**, with East Antarctica rotating **clockwise by 7.196°** relative to fixed West Antarctica. In other words, East Antarctica moved counterclockwise relative to the fixed West Antarctica with respect to the stage pole of 164.674°E and 68.880°S.

The confusion arose because rotation poles can be expressed in two mathematically equivalent ways: a pole in the Northern Hemisphere with clockwise rotation, or its antipodal point in the Southern Hemisphere with counterclockwise rotation (Figure R1). We inadvertently mixed these conventions in our presentation, creating the appearance of inconsistency. When properly applied, the pole at 344.674°E, 68.880°N with clockwise rotation of East Antarctica (identical to the pole at 164.674°E and 68.880°S with clockwise rotation of West Antarctica) produces exactly the ESE-directed motion shown in Figure 4 (now Figure 3).

The stage pole location in the northern North Pacific (344.674°E, 68.880°N) means that West Antarctica's motion relative to East Antarctica follows small circles around this pole. For the Central Basin region at approximately 70°S, 175°E, the geometry dictates that motion vectors point ESE, not ENE. This is because the Central Basin lies almost antipodal to the rotation pole, and the angular velocity decreases sinusoidally with distance from the pole, producing the ESE-–directed motion we observe (DeMets et al., 2010).

To verify this, we calculated motion vectors at multiple points across the Central Basin using the correct rotation parameters, and all consistently show ESE-directed motion with magnitudes of 7–8 mm/yr, precisely matching our magnetic anomaly constraints and the reconstruction shown in Figure 3.

Regarding the reviewer's comment about Figure 3 (now Figure 4), we respectfully disagree that it shows nothing new. While fracture zones west of Balleny FZ indeed record Australia–East Antarctica motion, our new contribution is demonstrating that the Balleny FZ flowline itself aligns with our Chron 24–20 stage pole. This alignment explains why the mismatch doesn't grow from Chron 24 to 20 despite ongoing East–West Antarctic motion—the motion was parallel to the fracture zone during this period. The synthetic flowlines in Figure 3 (now

Figure 4) diverge progressively from Chron 24 to 8, with maximum divergence near Victoria Land, exactly as predicted by our rotation parameters. This independent validation from fracture zone geometry strongly supports our Central Basin interpretation.

I laid out above the major issues of the manuscript, as there is no point in giving detailed comments on it before it is revised.

We thank the reviewer for these detailed and constructive comments that have significantly improved our manuscript. We have carefully addressed each of the major issues raised, including: (1) presenting a more thorough analysis of the Central Basin with all available data, (2) providing stronger justification for the asymmetric spreading interpretation, (3) demonstrating how the Balleny FZ test validates rather than contradicts our model, (4) clarifying the rotation pole parameters and their uncertainties, and (5) resolving the apparent directional inconsistencies through clearer presentation of our rotation conventions. We hope that our comprehensive revisions and additional evidence have addressed your concerns and demonstrate that the Central Basin provides compelling evidence for extending East-West Antarctic motion back to ~53 Ma. We believe the revised manuscript now presents this important finding with the rigor and clarity it deserves.

Briais, A., Patriat, P. & Tapponnier, P. Updated interpretation of magnetic anomalies and seafloor spreading stages in the south China Sea: Implications for the Tertiary tectonics of Southeast Asia. *J. Geophys. Res.* **98**, 6299–6328 (1993).

Brune, S., Heine, C., Pérez-Gussinyé, M. & Sobolev, S. V. Rift migration explains continental margin asymmetry and crustal hyper-extension. *Nat Commun* **5**, 4014 (2014).

Cande, S. C., Stock, J. M., Müller, R. D. & Ishihara, T. Cenozoic motion between East and West Antarctica. *Nature* **404**, 145–150 (2000).

Davey, F. J., Cande, S. C. & Stock, J. M. Extension in the western Ross Sea region-links between Adare Basin and Victoria Land Basin. *Geophysical Research Letters* **33**, 2006GL027383 (2006).

DeMets, C., Gordon, R. G. & Argus, D. F. Geologically current plate motions. *Geophysical Journal International* **181**, 1–80 (2010).

Granot, R., Cande, S. C., Stock, J. M. & Damaske, D. Revised Eocene-Oligocene kinematics for the West Antarctic rift system. *Geophysical Research Letters* **40**, 279–284 (2013).

Hellinger, S. J. The uncertainties of finite rotations in plate tectonics. *J. Geophys. Res.* **86**, 9312–9318 (1981).

Kirkwood, B. H., Royer, J.-Y., Chang, T. C. & Gordon, R. G. Statistical tools for estimating and combining finite rotations and their uncertainties: Statistical tools for plate reconstructions. *Geophysical Journal International* **137**, 408–428 (2002).

Naliboff, J. & Buiter, S. J. H. Rift reactivation and migration during multiphase extension. *Earth and Planetary Science Letters* **421**, 58–67 (2015).

Taylor, B., Goodliffe, A. M. & Martinez, F. How continents break up: Insights from Papua New Guinea. *J. Geophys. Res.* **104**, 7497–7512 (1999).

Tikku, A. A. & Cande, S. C. The oldest magnetic anomalies in the Australian-Antarctic Basin: Are they isochrons? *J. Geophys. Res.* **104**, 661–677 (1999).

Responses to Reviewer #2:

Additional evidence is needed to support the results proposed by the authors. Particularly, they are modeling magnetic anomalies that they surveyed on two lines in the central basin between the Hallett Ridge and the Iselin Bank. It is true, as they mentioned, that nobody has identified specific magnetic reversals in this region.

However there is previous data in this region already, so it needs to be taken into account. There are survey lines such as NBP9702 that measured magnetic anomalies and collected multibeam bathymetry. Some of those lines are partly or completely within the same region being discussed with the models shown here. There might even be other surveys but I did not check in detail for any more.

Now that the authors of the current manuscript have collected more data that could complement the lines already collected, they should include the results that were already measured, to make sure that the magnetic observations from the previous cruises are consistent with the interpretations being made in this manuscript.

This would probably require a modification to Figure 2 showing one or more additional previous survey lines and how they agree with the model being proposed here.

We thank the reviewer for highlighting the importance of incorporating previous data, particularly from cruise NBP9702. We fully agree and have substantially revised Figure 2 to demonstrate that **NBP9702 data not only support but strengthen our interpretation**.

The revised Figures 2a and 2b now display the actual magnetic wiggles from the NBP9702 cruise that partly crossed into the Central Basin (shown as gray lines), allowing direct visual comparison with our new data. This comparison demonstrates clear consistency in both wavelength and polarity sequence between the datasets. While magnetic data from NBP9702 within the Central Basin have existed in the literature (Cande et al., 2000), they were not previously used to explain the basin's tectonic history due to the lack of complete transects and definitive age constraints. Our new survey now enables these existing NBP9702 picks to be properly interpreted as part of the Chron 24–20 sequence, and this independent agreement strongly validates our forward modeling results shown in Figures 2c and 2d.

Importantly, the magnetic isochrons previously interpreted from NBP9702 could not constrain the Central Basin's extension because they do not fully traverse the basin or adequately sample its spreading fabric. Thus, while NBP9702 captured partial magnetic evidence of basin formation over 20 years ago, this evidence remained uninterpreted until our complete transects provided the necessary framework. This limitation makes our new survey essential for resolving the tectonic history of the Hallett Ridge and Iselin Bank.

We confirm that NBP9702 is the only previous cruise in public databases with magnetic profile data from the Central Basin vicinity. All available data have been incorporated in our analysis and are represented in Figures 2a and 2b by different symbols: squares for Cande et

al. (2000), circles for Davey et al. (2006), and triangles for Granot et al. (2013), with colors indicating their corresponding geomagnetic Chrons. **Our work therfore not only provides new critical data but also gives proper tectonic context to previously orphaned magnetic observations**.

Cande, S. C., Stock, J. M., Müller, R. D. & Ishihara, T. Cenozoic motion between East and West Antarctica. *Nature* **404**, 145–150 (2000).

Davey, F. J., Cande, S. C. & Stock, J. M. Extension in the western Ross Sea region-links between Adare Basin and Victoria Land Basin. *Geophysical Research Letters* **33**, 2006GL027383 (2006).

Granot, R., Cande, S. C., Stock, J. M. & Damaske, D. Revised Eocene-Oligocene kinematics for the West Antarctic rift system. *Geophysical Research Letters* **40**, 279–284 (2013).

<u>Responses to Reviewer #3</u>:

Review of the manuscript "Revising the tectonic chronology of East-West Antarctica since the breakup of East Gondwana" by Choi et al. for publication in Nature Communications.

The manuscript presents a new magnetic survey from the Central Basin and subsequently proposes a tectonic model to account for misfits in earlier reconstructions.

The manuscript is well written, has high quality figures and the data supports the presented model.

I have to admit that I am not an expert in magnetic surveying, but as a paleomagnetists, I am always impressed by the detail possible in reconstructions using magnetic anomalies. I enjoyed reading the manuscript, which apparently significantly improved our understanding for this complex area.

My only comment would be that the begin of the introduction is a bit misleading. Is Antarctica important due to a unique position or due to a unique tectonic evolution? Is the interaction of two part in a large continent unique, or is Antarctica so interesting because it is so inaccessible? Of course, the position and the initiation of the circum-Antarctic flow is very important for the climate, but the current paper is not really about that, right? So I am just suggesting to scale down the introduction a bit, not to appear too exaggerating.

We thank the reviewer's positive assessment of our manuscript and for this constructive comment about the introduction. We agree that the original introduction may have overemphasized aspects of Antarctic geology that are not directly relevant to our study. Thus, we have revised the introduction to focus more specifically on the tectonic aspects that are pertinent to our research as follows:

"The Antarctic Plate serves as a critical reference frame for global plate tectonic reconstructions, being the only major plate surrounded entirely by active spreading centers (Gordon, 2000). The interactions between East and West Antarctica–two distinct geological provinces that functioned as separate plates during much of the Cenozoic–directly impact our understanding of Southwest Pacific plate motions and the breakup chronology of East Gondwana. Marine magnetic anomalies in the Ross Sea preserve the record of East-West Antarctic separation, yet the timing and kinematics of early motion remain controversial. Resolving this tectonic history is essential for removing persistent gaps in plate reconstruction models between the Lord Howe Rise and Campbell Plateau, which have implications for understanding regional tectonics and basin formation throughout the Southwest Pacific."

Gordon, R. G. The Antarctic connection. *Nature* **404**, 139–140 (2000).

Responses to Reviewer #1:

The revised version of the manuscript shows some improvement compared to the initial submission; however, the fundamental issues that undermined the original paper remain unresolved. Consequently, I regret to say that I cannot recommend the manuscript for publication in Nature Communications. My main concerns are outlined below.

We sincerely appreciate the reviewer's continued engagement with the manuscript. The reviewer's comments have led to substantial improvements, including the systematic comparison of symmetric and asymmetric models and the comprehensive presentation of all available magnetic profiles. We address each concern below.

While the study presents new data from the Central Basin, the interpretations and claims made by the authors are not convincingly supported by these data. In response to my previous concern regarding the proposal of one-sided seafloor spreading in the Central Basin—an observation unprecedented anywhere—the authors argue that the conjugate crust possibly lies between the Adare Basin and Cape Adare, where permanent ice prevents magnetic data acquisition. They therefore describe this region as poorly constrained. However, this characterization is inaccurate. The area has been extensively surveyed, most notably through the dense aeromagnetic grid collected during the German GANOVEX IX project (Damaske et al., 2007). These data clearly show magnetic lineations that formed as part of the Southeast Indian Ridge (SEIR) spreading system between Australia and East Antarctica. Thus, the assertion that this crust belongs to the Central Basin spreading system is not supported. In my view, the tectonic interpretation of the Central Basin presented here remains premature and requires substantially more data and analysis before a robust geodynamic model can be proposed.

We thank the reviewer for raising the GANOVEX IX aeromagnetic data (Damaske et al., 2007). We were aware of this dataset and its extensive coverage west of the Adare Trough, but in our previous submission we were not confident in using it as negative evidence for conjugate picks, as the GANOVEX IX project was not designed to examine the Central Basin case.

The reviewer's assessment that the GANOVEX IX magnetic lineations are attributable to the Southeast Indian Ridge (SEIR) system is an important clarification. We now accept this interpretation, and it has a constructive implication for our study: if the area west of the Adare Trough is confirmed as SEIR-generated crust, then conjugate magnetic anomalies from Central Basin spreading — if they exist — must reside within the Central Basin itself rather than farther west. Accordingly, in the revised manuscript we removed the speculative question marks that previously indicated predicted conjugate locations outside the basin (cf. previous Fig. 2a) and instead focused all magnetic anomaly identification within and adjacent to the Central Basin proper.

To strengthen the observational basis, we now present magnetic anomaly picks from all available profiles that cross the Central Basin, including five NBP9702 lines and our two new Araon survey lines (revised Fig. 2 and new Fig. 3). Across these seven profiles, the C20–C24 anomaly sequence is consistently identified, with ages increasing eastward from the Hallett

Ridge toward the Iselin Bank. This consistency across independent datasets provides robust constraints on the Central Basin's formation history regardless of whether the spreading was asymmetric or symmetric.

Regarding the spreading mechanism, we agree with the reviewer and the editor that our data alone cannot definitively exclude symmetric extension. Therefore, we conducted systematic forward modeling of both symmetric and asymmetric scenarios (revised Fig. 2a). Although asymmetric extension yields lower average residuals for both survey lines, we now present it as our preferred hypothesis and discuss symmetric extension as a viable alternative, as requested by the editor. We note, however, that the symmetric model requires the Central Basin to have acted as a micro-plate or to have undergone ridge jumps, in order to reconcile internally inconsistent spreading rates between the northern and southern profiles — a condition that is less plausible at the observed low spreading rates (~7–9 mm/yr).

The remainder of the paper is based on a global plate kinematic reconstruction (basically not using the new data), similar in approach to several prior studies (e.g., Cande and Stock, 2004). The derived rotation parameters are characterized by very large uncertainties, yet these uncertainties are not appropriately accounted for in the resulting reconstructions. This raises a fundamental question about the added value and novelty of the present work relative to existing literature.

We respectfully address the concern that our work offers limited novelty relative to existing plate circuit studies. Based on the existence of a gap between the Lord Howe Rise and Campbell Plateau in Chron 27 reconstruction, prior studies (e.g., Cande & Stock, 2004) *inferred the possibility* of pre-Chron 20 East–West Antarctic motion, but lacked direct observational evidence from the intervening plate boundary. The Central Basin magnetic data presented here — now corroborated by all available NBP9702 profiles (new Fig. 3) — provide the first direct constraint on this motion. This observational foundation is the principal new contribution, not the plate circuit methodology itself.

We acknowledge that the rotation parameters for Chrons 24 and 20 carry large uncertainties, which is inherent to incipient plate motion with small total displacement estimated through indirect plate circuits. In the revised manuscript, we present these uncertainties explicitly and discuss their implications for the reconstruction. Despite these uncertainties, the rotation parameters are independently validated by the Balleny Fracture Zone mismatch analysis: the plateau in misfit distance between Chrons 24 and 20 — first documented by Cande et al. (2000) but not explained — is consistent with East–West Antarctic motion parallel to the fracture zone during this interval (Fig. 5, inset). This independent validation demonstrates that the rotation parameters, while uncertain in absolute terms, capture the correct sense and approximate magnitude of motion.

<u>Responses to Reviewer #2</u>:

The revised manuscript does an excellent job of responding to the reviewers' concerns and makes an excellent case for the arguments concerning the tectonic history.

The methodology is sound and the inclusion of the additional pre-existing data strengthens the validity of the results.

The figures have been modified to show clearly how all of the data are consistent with the proposed tectonic scenario.

I do not think any further changes are needed in order for this manuscript to be accepted for publication

We appreciate the reviewer's generous evaluation. Thank you.

Responses to Reviewer #4:

Review of "Revising the tectonic chronology of East–West Antarctica since the breakup of East Gondwana".

This paper sets out a revised tectonic history for the Central Basin between Hallett ridge and Iselin Bank in the Ross Sea. The revised history is based on new and existing magnetic data interpreted to reveal oceanic magnetic anomalies dated to between 53 and 43 Ma. Unusually the authors suggest extreme asymmetric spreading, explaining why only crust and associated anomalies accreted to the West Antarctic (Iselin Bank) side are preserved.

As a late reviewer I found the manuscript generally well written, and think that this work has the potential to provide a useful new hypothesis for the early evolution of West Antarctica. I do however, have a few concerns with the manuscript.

The paper stands or falls on the interpreted ages of the magnetic anomalies within the Central Basin. It would therefore be good to have a more complete description of why anomalies C20 and C24 are identified. The model fits relatively well (Fig. 2c and d), but could symmetrical spreading within the Central Basin work just as well, with the magnetic high in the basin centre reflecting a former spreading ridge, with approximate symmetric anomalies each side? A simple ridge jump into the nascent Adare Trough west of Hallett Ridge could then explain the basin, rather than requiring such extreme asymmetric spreading. This alternative hypothesis requires re-dating of the anomaly pattern, but might form a viable alternative hypothesis.

We thank the reviewer for this constructive comment, which addresses a central question of the manuscript. We respond to the two aspects in turn.

Identification of C20 and C24. In the revised manuscript, we strengthened the basis for identifying C20 and C24 by presenting magnetic anomaly picks from all available profiles that cross the Central Basin, including five NBP9702 lines and our two new Araon survey lines (new Fig. 3). Across these profiles, the C20 pick is consistently identified over or near the Hallett Ridge at the western margin of the basin, and the C24 pick is identified near the Iselin Bank at the eastern margin. The anomaly sequence C21 through C24 is recognized as a diagnostic sequence of 3–4 positive pulses, a pattern also documented in similar-age crust of the Australian–Antarctic Basin (Tikku & Cande, 1999). The identification of C24 as the oldest anomaly is further constrained by regional context: the adjacent Hallett Ridge and Iselin Bank were positioned near Cape Adare by Chron 27, and Central Basin formation postdates their emplacement, placing a lower bound on basin age. The identification of C20 as the youngest anomaly is consistent with the onset of Adare Trough spreading at Chron 20 (Cande et al., 2000), which would have terminated Central Basin extension. These boundary conditions, combined with the forward modeling fit (revised Fig. 2a), provide robust constraints on the C20–C24 age assignment.

Symmetric vs. asymmetric extension. Following the reviewer's suggestion, we conducted systematic forward modeling of both symmetric and asymmetric extension scenarios for both survey lines (revised Fig. 2a: Models N1–N4 and S1–S4). In the symmetric models, the

spreading axis is placed at or near the bathymetric/magnetic high within the Central Basin, and conjugate anomalies are modeled on both sides. In the asymmetric models, anomalies accrete only to the eastern (West Antarctic) side. For Line-N, the asymmetric model (N3) yields the lowest average residual. For Line-S, S3 and the symmetric C27–C24 model (S2) produce comparable residuals; however, the C27–C24 timeframe is inconsistent with our anomaly identification (see Fig. 3). When both lines are considered jointly, the asymmetric C24–C20 models (N3, S3) provide the best overall fit.

We further note that the symmetric model produces internally inconsistent spreading rates between the northern and southern profiles: the asymmetry in rates between the two flanks reverses direction from one line to the other (Fig. 2a). To reconcile this inconsistency, the symmetric scenario would require the Central Basin to have acted as an independent microplate or to have undergone intra-basin ridge jumps — conditions that are difficult to sustain at the observed low spreading rates (~7–9 mm/yr). In addition, ridge jumps typically produce pseudofaults in the magnetic anomaly pattern, for which we find no evidence in the available data.

Beyond forward modeling, we now present the tectonic reconstruction implications of both models side by side in revised Figure 4. The symmetric reconstruction (Figs. 4a–c) employs Cande & Stock (2004)'s C27–C24 rotation parameters repurposed for the C24–C20 interval, as no independent parameters exist for this period under their framework. These borrowed parameters cannot be independently validated through the global plate circuit for the C24–C20 interval. The asymmetric reconstruction (Figs. 4d–f) is directly constrained by C24o and C20o, the bounding isochrons identified in the Central Basin, enabling derivation of new rotation parameters through the East Antarctic–Australian–West Antarctic plate circuit (Table 1). These parameters are independently testable — a capability the symmetric model lacks. We therefore consider asymmetric extension the preferred hypothesis, while acknowledging the symmetric alternative.

Associated with this point, in Figure 2b there are 4 marine magnetic picks attributed to Cande et al 2000 south of ~70.5 S within the Central Basin. Looking at the Cande et al 2000 paper (their Fig. 3) I could not find these points. Am I misunderstanding the Cande et al 2000 paper, or did the authors identify these points in older magnetic datasets attributed to Cande et al 2000? Clarity on this is important as if previously identified as dated anomalies they stand as an independent check on the identification of C20 and C24 within the Central Basin. If not, it should be clarified that they form part of the authors' interpretation.

We thank the reviewer for identifying this ambiguity, and we apologize for the confusion. The four magnetic picks south of ~70.5°S within the Central Basin (shown in Fig. 2b of the previous submission) were *newly identified by the authors* from NBP9702 magnetic data, not picks published in Cande et al. (2000). These picks were attributed to Cande et al. (2000) in our figure because the NBP9702 cruise was conducted as part of their study, but they were not identified or published in that paper.

In the revised figures, we clarified this distinction. Picks previously published in the literature retain their original source symbols (squares for Cande et al., 2000; circles for Davey et al., 2006; triangles for Granot et al., 2013), while picks newly identified by the authors — whether from NBP9702 data or from our Araon survey — are shown as star symbols (open stars for NBP9702-derived picks; filled stars for new Araon data). The figure legend and text have been updated accordingly to prevent any misattribution.

I also have some concerns about the ambiguity about what Hallet Ridge is (oceanic or continental fragment). On one hand it is argued that it is oceanic, containing anomaly C20 (~43 Ma), (L128 to 132). However, the authors subsequently state L154 "Our reconstruction model suggests that the Iselin Bank in the northern Ross Sea was positioned adjacent to the Hallett Ridge at Chron 24 (~53 Ma), immediately prior to Central Basin formation", and see Fig. 3. These two statements appear contradictory, if Hallett Ridge is 43 Ma oceanic crust, it can't have been present adjacent to the Iselin Bank at 53 Ma. It may be that Hallett Ridge is a composite feature – looking at the bathymetry in detail shows that the broader southern HR appears as an extended shallow (2000-1600 m depth) province. This could reflect fragments of continental crust within a developing oceanic marginal basin, with anomaly C20 developing at/within the margin, which is preserved as Hallett Ridge. I would advise expanding the discussion of the origin of the ridge to acknowledge this ambiguity, and also to propose if there are other observations which could be used to test this hypothesis in future? An alternative model where Hallett Ridge was a fragment of the continental margin isolated by a ridge jump into the Adare Basin could also be proposed.

We thank the reviewer for this perceptive observation. The reviewer is correct that a contradiction exists in our previous text: if the Hallett Ridge were entirely C20 oceanic crust (~43 Ma), it could not have been positioned adjacent to the Iselin Bank at Chron 24 (~53 Ma). We accept the reviewer's interpretation that the Hallett Ridge is likely a composite feature, and we have revised the manuscript accordingly.

As the reviewer notes, the bathymetry of the broader southern Hallett Ridge shows an extended shallow province (2,000–800 m depth) that is distinct from the narrower northern segment. We now interpret this as follows: the Hallett Ridge likely comprises continental crustal fragments that were originally part of the East Antarctic margin, within which C20 oceanic crust developed at or near its eastern margin during the final stage of Central Basin spreading. Under this interpretation, the pre-existing continental portion of the Hallett Ridge was indeed adjacent to the Iselin Bank at Chron 24, and the C20 magnetic signature records the youngest phase of extension before the spreading axis migrated westward into the Adare Trough.

This composite origin also accommodates the alternative model suggested by the reviewer, in which the Hallett Ridge was a fragment of the continental margin subsequently isolated by a ridge jump into the Adare Basin. We discuss both possibilities in the revised manuscript and note that they are not mutually exclusive—the continental fragments may have been progressively isolated as the extensional locus shifted. Importantly, this composite

interpretation is consistent under both the symmetric and asymmetric reconstruction models now presented in Figure 4. Under the symmetric model (Figs. 4a–c), the Hallett Ridge was part of the East Antarctic Plate during the Central Basin spreading phase and was subsequently transferred to the West Antarctic Plate when the extensional axis migrated to the Adare Trough. Under the asymmetric model (Figs. 4d–f), the Hallett Ridge remained relatively fixed during this interval as new crust accreted primarily toward West Antarctica. Under both models, the ridge was ultimately isolated from East Antarctica at Chron 20.

We further propose that the following observations could test the composite-origin hypothesis in future studies: (1) seismic refraction data across the Hallett Ridge could resolve whether the deeper crustal structure is continental or oceanic; (2) dredge sampling or drilling of the ridge could constrain its basement lithology; and (3) higher-resolution aeromagnetic surveys could determine whether the C20 signature is confined to the eastern margin of the ridge or extends across its full width. We have incorporated these points in the revised Discussion.
